# A qPCR method for genome editing efficiency determination and single-cell clone screening in human cells

**DOI:** 10.1038/s41598-019-55463-6

**Published:** 2019-12-11

**Authors:** Bo Li, Naixia Ren, Lele Yang, Junhao Liu, Qilai Huang

**Affiliations:** 1 Shandong Provincial Key Laboratory of Animal Cell and Developmental Biology, School of Life Sciences, Shandong University, Qingdao, China; 2 State Key Laboratory of Microbial Technology, Shandong University, Qingdao, China; 3 The Second Hospital of Shandong University, Jinan, China

**Keywords:** Synthetic biology, PCR-based techniques

## Abstract

CRISPR/Cas9 technology has been widely used for targeted genome modification both *in vivo* and *in vitro*. However, an effective method for evaluating genome editing efficiency and screening single-cell clones for desired modification is still lacking. Here, we developed this real time PCR method based on the sensitivity of Taq DNA polymerase to nucleotide mismatch at primer 3′ end during initiating DNA replication. Applications to CRISPR gRNAs targeting *EMX1*, *DYRK1A* and *HOXB13* genes in Lenti-X 293 T cells exhibited comprehensive advantages. Just in one-round qPCR analysis using genomic DNA from cells underwent CRISPR/Cas9 or BE4 treatments, the genome editing efficiency could be determined accurately and quickly, for indel, HDR as well as base editing. When applied to single-cell clone screening, the genotype of each cell colony could also be determined accurately. This method defined a rigorous and practical way in quantify genome editing events.

## Introduction

CRISPR/Cas9 has become the major genome-editing technology and been widely used in different kinds of organisms for genome modification purpose^1–4^. In the CRISPR/Cas9 system, Cas9 nuclease is directed to target DNA containing the protospacer adjacent motif (PAM) by single guide RNA (sgRNA), then cleaves both strands of target DNA at a site 3 bp upstream of the PAM sequence and generates double-strand breaks (DSBs). This kind of DNA breaks is harmful to cells and can lead to mutagenesis or cell death if left unrepaired. Once sensed, the DSBs will be repaired mostly by two different kinds of intrinsic mechanisms, homology-directed repair (HDR) or non-homologous end joining (NHEJ)^5,6^. The former relies on a homologous sequence as the repair template and repairs DNA breaks in a high-fidelity manner. It is usually employed to introduce specific DNA modifications, to meet the needs in functional study of genetic variation, especially in clinical usage. The latter involves direct ligation of the broken ends without the need for a homologous template and repairs DNA breaks in an error-prone manner. The NHEJ usually leads to unpredictable insertion or deletion of bases in the genome, named indel, which will most likely disrupt the open reading frame of target gene. This makes it a very effective method in destroying gene expression for gene functional study and in clinical to remove pathogenic genes^7,8^.

Usually, for any experimental purpose prescreening the sgRNAs for high editing efficiency and specificity is essential and screening the single-cell clones or offspring bearing desired modification events are often obligatory. The present techniques for evaluating genome editing efficiency have been well discussed and compared in a review^9^. The widely used methods are mainly based on DNA sequencing or mismatch-specific nuclease^9,10^. Sanger sequencing method involves PCR amplification and cloning steps of the target region before each DNA sequence being read separately. This multistep method can provide detailed information of each mutation event induced by nuclease, but is quite time-consuming, costly and laborious^10^. To overcome these disadvantages, computational algorism was introduced to realize editing efficiency quantification based on direct Sanger sequencing of amplicon mixture of target DNA region. Whereas its reliability tends to be impeded by repetitive sequence around the cutting site and highly depends on the purity of PCR product and the quality of Sanger sequencing^11^. The next-generation DNA sequencing (NGS) technology was also applied in profiling DNA mutation induced by sgRNA directed Cas9 nuclease owing to its massive parallel capacity^12^. Several web-based online platforms have been developed to analyze the NGS data, including CRISPR-GA^13^, BATCH-GE^14^, CRISPResso^15,16^, Cas-analyzer^17^ and CRISPRMatch^18^ *et al*. However, even though effective, these NGS-based methods still require multi-step operations and are costly in time and money. The mismatch specific nuclease-based methods employ T7 endonuclease 1 (T7E1) or Surveyor nuclease to cleave mismatches formed between DNA strands containing sequence difference originated from nuclease cutting^19^. They require only basic laboratory equipment but not applicable to polymorphic loci and tend to miss single-nucleotide mutation as well as large deletions^20^. In addition, many other alternatives have been developed with improvement in certain aspects, including qEva-CRISPR^21^, engineered nuclease-induced translocations(ENIT)^22^, Cas9 nuclease based restriction fragment length polymorphism (RFLP) analysis^23^, Indel Detection by Amplicon Analysis (IDAA)^24^ and the gene-editing frequency digital PCR (GEF-dPCR)^25^. However, most methods are multistep and quantify the editing efficiency based on pre-amplified PCR product coming from genomic DNA but not directly on the genomic DNA itself^9–20,22–24^. Sequence and length-dependent bias introduced during PCR amplification will unavoidably affect the detection accuracy^26–28^. Moreover, many methods demand specific devices, such as capillary electrophoresis apparatus^21,24^, digital PCR system^25^ and NGS platform^12–18^ that are expensive and not readily available in most laboratories. As for offspring genotyping and single-cell clone screening, besides Sanger sequencing and NGS based methods^29,30^, several other strategies have also been developed specifically for genotyping purpose including high-resolution melting (HRM)^31^ and oligoribonucleotide interference-PCR (ORNi-PCR)^32^ *et al*. In zebrafish^33^ and plant^34^, PCR based methods have been developed for mutant screening, but limited accuracy and sensitivity restricted its wide applications.

Here we developed a real-time PCR based method, namely genome editing test PCR (getPCR) by combining the sensitivity of Taq polymerase to mismatch at primer 3′ end with real-time PCR technique for its power in DNA quantification. Applications in Lenti-X 293 T cells on 9 sgRNA targets indicate that this technique could determine the genome editing efficiency accurately in all cases of genome editing including NHEJ induced indels, HDR and base editing. Meanwhile, this method exhibited great power in single-cell clone genotyping by its ability in telling exactly how many alleles were modified. This technique described here provides the most robust strategy by far that can be used not only in genome editing efficiency quantification but also single-cell clone genotyping in a high throughput way.

## Results

### Principle of getPCR method

**Figure 1:**
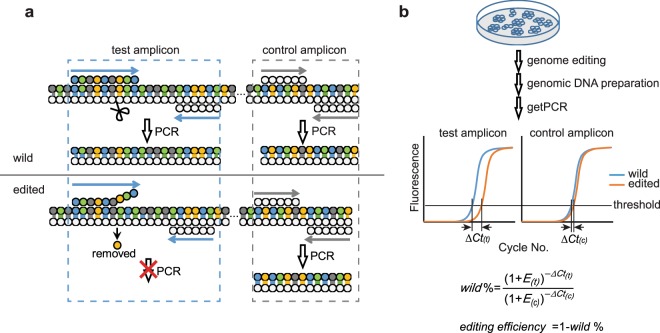
Principle and flowchart of getPCR. (**a**) Principle of getPCR in discriminating indel and wild sequences. (**b**) Overview of getPCR strategy.

Efficiency evaluation of CRISPR gRNA means basically the calculation of indel frequency after genome editing takes place. Real-time PCR technology is the most powerful method in nucleic acid quantification. However, the multiplicity and unpredictability of indel profile make it impossible to design indel specific primers and consequently impractical to quantify the indel frequency directly through real-time PCR. The getPCR technology bypasses this obstacle by quantifying the proportion of un-edited wild sequence first and hence realize the determination of genome editing efficiency indirectly. This method utilizes the sensitivity of Taq DNA polymerase to mismatch at the primer 3′ end^35^. The watching primer that is responsible for discriminating indel modifications is designed to span Cas9 nuclease cutting site near its 3′ end to render getPCR the ability in selectively amplifying wild type sequence only **(**Fig. 1a**)**. More importantly, a control amplification is introduced hundreds base pairs away from the cutting site for normalization purpose in calculating the wild type DNA percentage in the edited genomic DNA sample by a ∆∆C_t_ strategy **(**Fig. 1b**)**. Then, the editing efficiency of given sgRNA can be determined finally. When applied to single-cell colony genomic DNA samples, their genotypes can be defined easily.

### Watching primer design for getPCR

**Figure 2:**
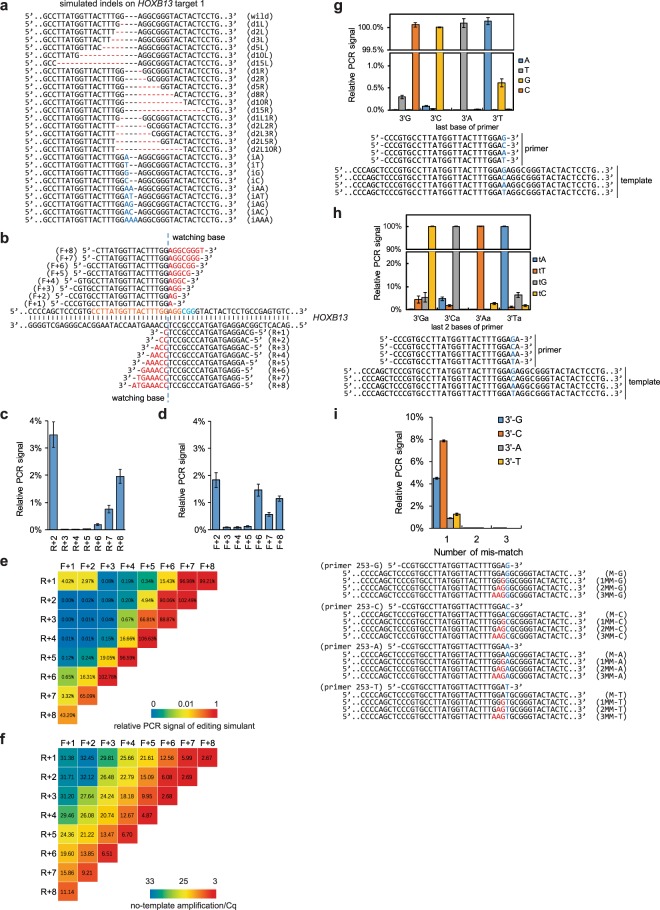
Principle of getPCR primer design. (**a**) Twenty-six plasmids constructed to mimic indels at *HOXB13* gene gRNA target 1. (**b**) Sixteen types of watching primers with different number of watching bases for getPCR detection of genome modifications at *HOXB13* gene gRNA target 1. (**c,d**) Evaluation of their ability in discriminating indels for reverse primers and forward primers respectively. Bar charts display relative PCR signals for indels to wild sequence. The lower signal reflects higher ability in discriminating indels. (**e**) Heatmap illustration of the combination effect of forward and reverse primes in discriminating indels. Lower relative amplification signals from indel template indicate higher ability in discriminating indels. (**f**) Investigation of the background self-amplification signal of partially overlapping watching primer pairs without template DNA. Heatmap displays the Ct value. Smaller Ct value indicates stronger background self-amplification. (**g**) Influence of the first base at primer 3′ end on PCR amplification specificity. Bar chart shows the relative PCR signal from matched or mismatched templates. (**h**) Effect of mismatch type at primer 3′ end second last position on PCR amplification efficiency. Bar chart shows the relative PCR signal from matched or mismatched templates. (**i**) Primer 3′ end base type affect sensitivity to neighbor mismatch. Bar chart shows the relative PCR signal from templates containing different kinds of mismatches. (Means ± s.e.m, n = 3 independent technical replicates).

To make getPCR technique work, the principle needs to be determined for designing watching primer which is responsible for discriminating indels from wild sequence. Given that most indels occur surrounding the nuclease cutting site and small indels less than 15 bps accounts for the major part^12,36^, and meanwhile, only small indels are supposed to be given extra concerns for discrimination, we designed 26 plasmid constructs representing 1–15 bp indels to mimic *in vivo* nuclease induced genome editing at sgRNA targeting *HOXB13* gene **(**Fig. 2a**)**. Two serials of primers with one to eight watching base(s) were designed **(**Supplementary Fig. 1a–c**)** and those with adequate amplification efficiency were chosen **(**Fig. 2b**)** for further examination of their ability in discriminating indels. Theoretically, more watching base could increase the selectivity of watching primer. However, too many watching bases will make the mismatch move away from the 3′ end to the 5′ end and consequently impede the sensitivity of Taq polymerase on the contrary. When single direction watching primer employed, 3 to 5 watching bases exhibited preferable distinguish ability of indel sequences from wild type sequence for both reverse **(**Fig. 2c**)** and forward **(**Fig. 2d**)** primers. When forward and reverse watching primers applied in combination, 4 to 6 watching bases in sum could discriminate indels successfully **(**Fig. 2e, Supplementary Fig. 1d**)**. However, 5 or 6 additive watching bases showed higher background signal because of primer self-amplification **(**Fig. 2f, Supplementary Fig. 1e**)**. Therefore, 4 additive watching bases are ideal for designing combinational getPCR primers.

The 3′ end base of watching primer plays substantial roles in determining getPCR discrimination ability. The adenine base displayed best specificity and gave lowest non-specific amplification signal when mismatched with non-complementary bases. Cytosine came the second followed by guanine and thymine **(**Fig. 2g**)**. When the mismatch located in the second last position, similar results were observed. The adenine base still displayed the best specificity and its mismatch with non-complementary bases was less tolerated by Taq polymerase **(**Fig. 2h**)**. In addition, the 3′ end base type also determined the sensitivity of getPCR to mismatch happened upstream. Again, adenine base is the best choice and enables PCR amplification most sensitive to mismatch happened at the second last position. It is worth noting that, if more than one mismatches occurred neighboring to the last base, the PCR amplification will be obviously destroyed whatever the last base is **(**Fig. 2i**)**. Moreover, the closer to the 3′ end the mismatch is, the more sensitive to the mismatch the getPCR becomes **(**Supplementary Figs. 1f,g, 2a-b**)**.

To explore the potential mechanisms that enable getPCR sensitive to mismatch, we compared the PCR amplification of 3′ end-mismatched primer with mismatch base-deleted primer. Interestingly, the deletion of mismatch base partially restored the amplification capacity in qPCR as well as common PCR analysis **(**Supplementary Figs. 1h-i, 2a,b**)**. Besides, high-fidelity DNA polymerases such as Phusion and Q5 that possess the proofreading 3′ to 5′ exonuclease activity could also restore the PCR amplification in part or completely. Sanger sequencing chromatograms of the PCR products showed that the mismatched nucleotide at the primer 3′ end was removed by the 3′ to 5′ exonuclease activity during polymerizing. On the contrary, Taq DNA polymerase without 3′ to 5′ exonuclease activity just tolerated and bypassed the mismatch directly **(**Supplementary Fig. 2c**)**. It indicates that, the mismatch impeded primer pairing with the template on one hand, and the spatial geometric hindrance caused by the mismatch further hampered Taq polymerase priming.

Briefly, for the watching primer design, 3, 4 and 5 watching bases are good choices to obtain reasonable indel discrimination ability. As for watching base type, adenosine is the best choice, followed by cytosine and guanine, whereas thymine should be avoided.

### Parameters for running a getPCR

**Figure 3:**
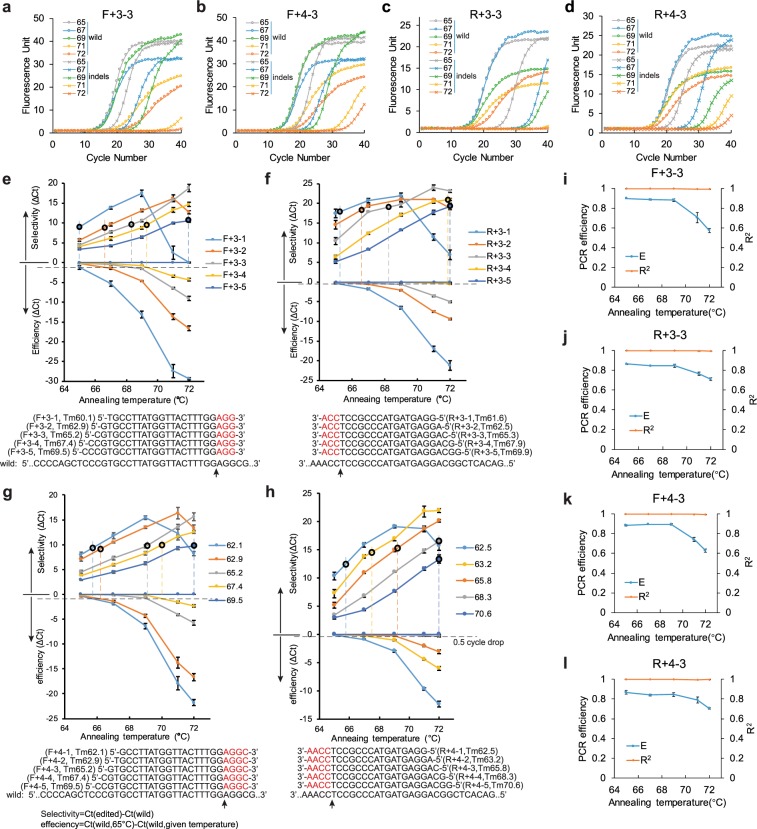
Parameter optimization for running getPCR. (**a,d**) Amplification curves of DNA templates with or without indels using four watching primers at different annealing temperature. The watching primers contain 3 (**a**) or 4 (**b**) watching bases in forward direction or 3 (**c**) or 4 (**d**) watching bases in reverse direction respectively. (**e–h**) Line charts showing the influence of watching primer’s Tm value on the PCR efficiency and selectivity over indels at different annealing temperature in PCR amplification, using forward watching primers with 3 (**e**) or 4 (**g**) watching bases and reverse watching primers with 3 (**f**) or 4 (**h**) watching bases. PCR efficiency is shown as ∆Ct calculated relative to Ct value at 65 °C and selectivity is shown as ∆Ct between wild type and indel templates. Watching primer sequences are shown in the bottom with watching bases highlighted in red. The small circle denotes the best selectivity under optimum amplification efficiency at 0.5cylce dropped Ct value as indicated by the dashed line. (**i-l**) Influence of annealing temperature on PCR amplification efficiency and the linearity of standard curve, characterized by R square value. Four watching primers employed in the examination are forward with three(**i**) or four(**k**) watching bases and reverse with three(**j**) or four(**l**) watching bases respectively. (Means ± s.e.m, n = 3 independent technical replicates).

The other issue needs to be addressed for getPCR is the optimum parameter, mainly annealing temperature in performing getPCR reaction. Using plasmids simulating indels at *HOXB13* target 1, getPCR primers with 3 or 4 watching bases were chosen to determine the optimal parameter, which is supposed applicable to getPCR primer containing 5 watching bases. Along with the elevation of annealing temperature, the amplification specificity for matched wild template over mismatched indel templates obviously increased for all the four watching primers **(**Fig. 3a–d**)**. However, when the annealing temperature increased to over 4 °C higher than Tm value, the PCR efficiency began to drop obviously on the contrary. Since optimal PCR efficiency is usually preferred for PCR amplification, the best selectivity of each watching primer was systematically evaluated under optimal PCR efficiency **(**Fig. 3e–h**)**. Intriguingly, no matter how many watching bases and total bases the primer had, the best selectivity was often observed at the annealing temperature about 4 °C higher than its Tm value **(**Fig. 3e–h**)**. With fixed watching base number, increasing primer Tm value by adding more bases at its 5′ end didn’t dramatically alter the ability in discriminating indels. Three of the four types of primers exhibited steady ability in discriminating indels **(**Fig. 3e–g**)**. Only one type of primer showed slightly increased ability and reached optimum at Tm value around 65.8 °C **(**Fig. 3h**)**. Therefore, in subsequent experiments watching primers were designed with Tm value around 65 °C and getPCR were performed with annealing temperature 69 °C for all kind of watching primers. More importantly, even though the increased annealing temperature over Tm value impeded the PCR efficiency, the basis of real-time PCR quantification, i.e., the linear correlation between the Ct value and logarithm template DNA quantity, was not affected at all for all the four types of primers **(**Fig. 3i–l**)**.

DNA polymerase plays essential roles in determining the discrimination ability of getPCR. Even though varying in performance, almost all tested commercial Taq products exhibited acceptable ability in discriminating indels from wild type sequence **(**Supplementary Fig. 1j**)**. However, when sensitivity to single-base mismatch was evaluated, two from nine SYBR green qPCR products showed less applicable performance **(**Supplementary Fig. 2d,e**)**. It is worth noting that high-fidelity DNA polymerases are not applicable in the getPCR method because their 3′ to 5′ exonuclease activity can remove the mis-match nucleotides at the primer 3′ end and hence erase its ability in discriminating indels.

### GetPCR determined editing efficiency faithfully in simulated genome editing

**Figure 4:**
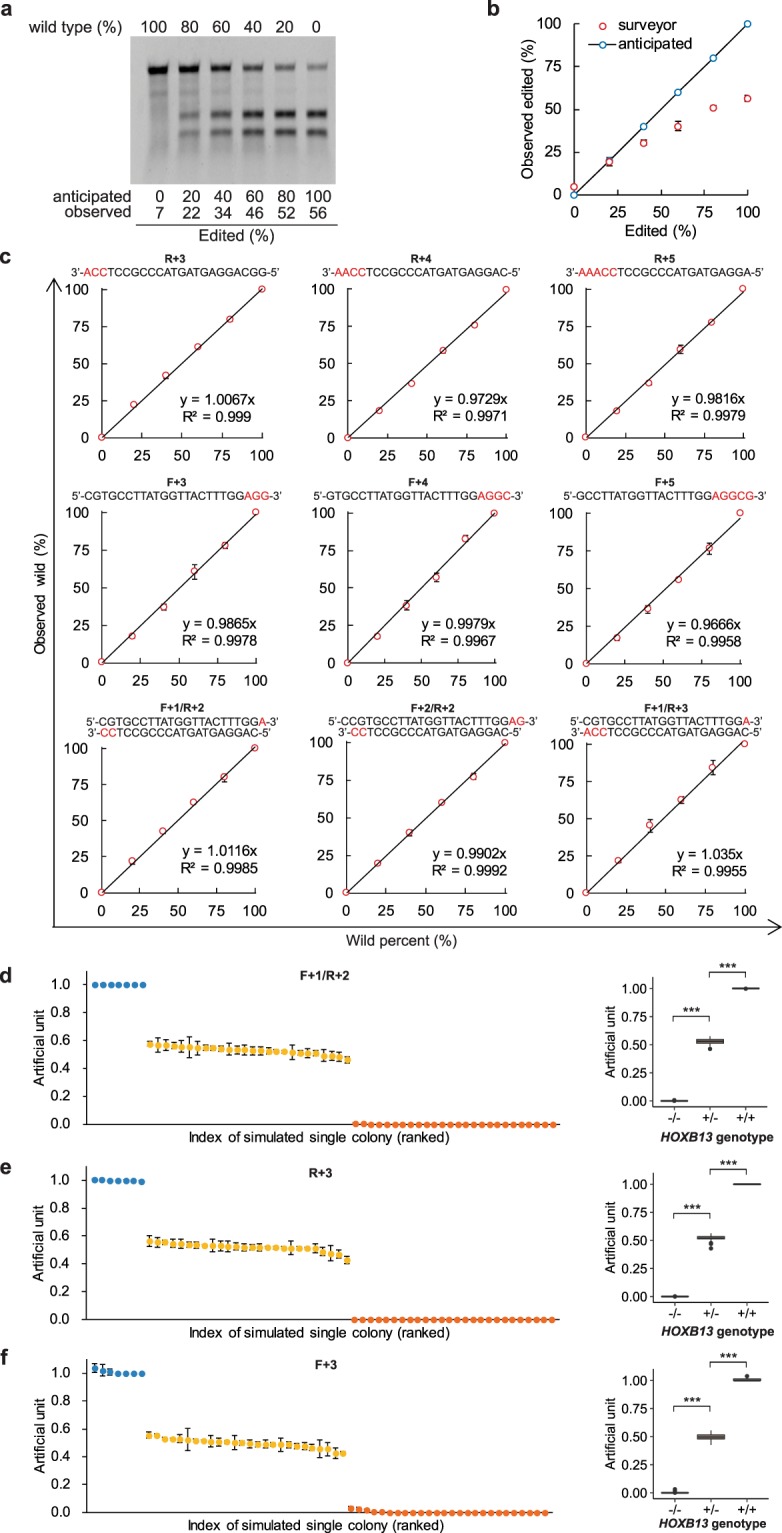
Application of getPCR in editing frequency determination and single-cell colony genotyping on indel-mimic plasmids. (**a**) Twenty-six plasmids simulating indels at *HOXB13* gene target 1 were mixed with wild type *HOXB13* plasmid at given ratios and evaluated with Surveyor assay method. (**b**) Apparent editing frequencies from quantified Surveyor assay results. (**c**) On the same indels mimics, indel frequencies were determined using getPCR method with forward and reverse watching primer alone or in combination. (**d–f**) Each of the 26 indel mimic plasmids were subjected to getPCR evaluation directly as double allele edited cell clones or equally mixed with wild type plasmid to simulate single allele edited cell clones. Three differently designed getPCR watching primers were tested, including F + 1/R + 2 (**d**), R + 3 (**e**) as well as F + 3 (**f**). (Means ± s.e.m, n = 3 independent technical replicates, *P < 0.05, **P < 0.01, ***P < 0.001).

The ability of getPCR in quantifying genome editing efficiency was first evaluated with plasmids simulating genome editing indels as used in Fig. 2a. Twenty-six plasmids with different indel mutations were combined equally and then mixed with wild construct at given ratio to mimic indel frequencies of 0%, 20%, 40%, 60%, 80% and 100%. The mixtures were subjected to indel frequency quantification by getPCR as well as the classic Surveyor method for comparison. When indel frequency is not higher than 20%, quantification results by Surveyor method could truly reflect the anticipated value. However, along with the further increasing of indel frequency, the observed value deviated from anticipated value progressively **(**Fig. 4a,b**)**. On the contrary, all the twelve getPCR strategies with different watching primers could accurately determine the indel frequencies **(**Fig. 4c, Supplementary Fig. 3a–c**)**. However, when indel frequencies decreased below 10%, the accuracy of getPCR method became limited gradually (Supplementary Fig. 3d).

### Application in genotyping of mimic single-cell clones

The getPCR technique can also be used in single cell clone screening or offspring genotyping in genome editing experiments. Each indel construct as shown in Fig. 2a alone or equally combined with wild construct was used to mimic single-cell clone genomic DNA with double alleles or one allele modified respectively. All the three getPCR strategies could accurately determine the genotypes of all the clones. Not only determine if indels happened, but also clarify how many alleles carried indels accurately **(**Fig. 4d–f**)**. In addition, when any two getPCR strategies were analyzed in combination, their detection values exhibited extremely high correlation with a Pearson Correlation Coefficient equal to or higher than 0.995. Intriguingly, the combination of two getPCR strategies could dramatically improve the performance in defining the genotype **(**Supplementary Fig. 3e–g**)**.

### Application of getPCR in indel detection in cells

**Figure 5:**
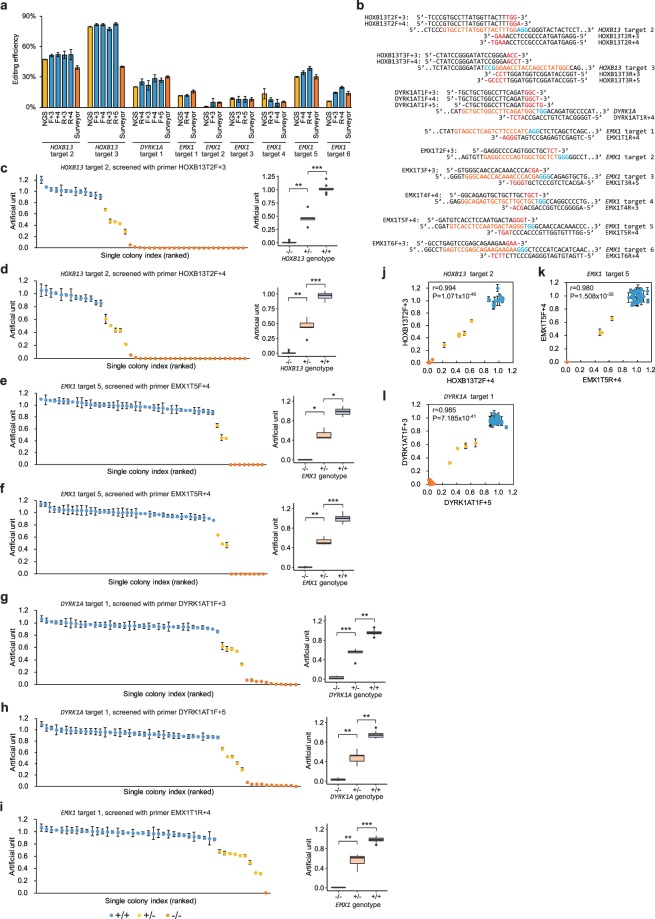
Indel frequency determination and single-cell colony genotyping in Lenti-X 293 T cells. (**a**) Application of getPCR in quantification of indel frequency generated at nine gRNA targets on *HOXB13*, *DYRK1A* and *EMX1* genes in comparison with NGS and Surveyor methods. (**b**) Illustration of gRNA sequences and watching primers employed in getPCR, with PAM sequence in blue, target sequence in orange and watching base in red. (**c–i**) Single-cell clones isolated and propagated from edited Lenti-X 293 T cells by high-fidelity version Cas9 nuclease with sgRNA targeting *HOXB13* gene (**c,d**), *EMX1* gene (**e,f,i**) and *DYRK1A* gene (**g,h**) were genotyped by getPCR methods. Box plots show quartiles with a band at median, whiskers indicating 1.5 IQR, and outliers shown separately. The correlation and combination effect of two differently designed watching primers were evaluated in genotyping(**j–l**). (Means ± s.e.m, n = 3 independent technical replicates, *P < 0.05, **P < 0.01, ***P < 0.001).

We applied getPCR in the detection of genome editing with high-fidelity Cas9 variant and nine different gRNAs targeting *HOXB13*, *DYRK1A* or *EMX1* gene in Lenti-X 293 T cells **(**Fig. 5b**)**. The Cas9 variant employs R661A/Q695A/Q926A to decrease off target cleavage^37^. The editing efficiency of each gRNA was determined by three different methods, including getPCR, NGS-based amplicon sequencing as well as Surveyor assay. The editing frequency determined by getPCR method was often comparable to the results from NGS method, which was believed to be the most reliable one. In contrast, the apparent editing frequency value determined by Surveyor method exhibited obvious deviation from the other two methods, especially at *HOXB13* target 2 and target 3 where the editing efficiencies were high **(**Fig. 5a**)**. The genome modified cells with gRNAs of *HOXB13* target 2, *EMX1* target 1 and 5 as well as *DYRK1A* target 1 were also isolated single-cell colony and propagated. The genomic DNA samples were prepared and subjected to genotyping by getPCR and verified through Sanger sequencing. Overall, all the single-cell clones from the genome editing experiments with these four gRNA targets were accurately genotyped by getPCR. Notably, not only the cell clones carrying indels could be detected, the one-allele modified cells and both-allele modified cells could be successfully identified at the same time **(**Fig. 5c–i, Supplementary Fig. 4a,b**)**. For genome editing performed at *HOXB13* gRNA target 2, 24 double allele-modified colonies and 5 single allele-modified colonies were accurately identified from total 42 colonies using two different designed getPCR primers containing 3 or 4 watching bases respectively **(**Fig. 5c,d, Supplementary Fig. 4h**)**. Similarly, for editing at *EMX1* target 5, 8 double allele-modified colonies and 3 single allele-modified colonies were identified by getPCR with primers carrying 4 watching bases, designed in forward or reverse direction. **(**Fig. 5e,f, Supplementary Fig. 4i**)**. As to *DYRK1A* gRNA target 1, from total 53 colonies 11 were defined to be double allele-modified and 5 to be single allele-modified using getPCR with four different designed watching primers, which carrying 3, 4, or 5 watching bases in forward direction or 4 watching bases in reverse direction respectively **(**Fig. 5g–h, Supplementary Fig. 4a,b,j**)**. For *EMX1* gene target 1, getPCR using the 4-watching base primer successfully identified 1 double allele-modified cell clone and 9 single allele-modified cell clones from 45 clones **(**Fig. 5i, Supplementary Fig. 4k**)**. Notably, any two differently designed getPCR exhibited highly correlated detection value and could help the genotyping when analyzed in combination **(**Fig. 5j–l, Supplementary Fig. 4c–g**)**.

### Application of getPCR in HDR detection in cells

**Figure 6:**
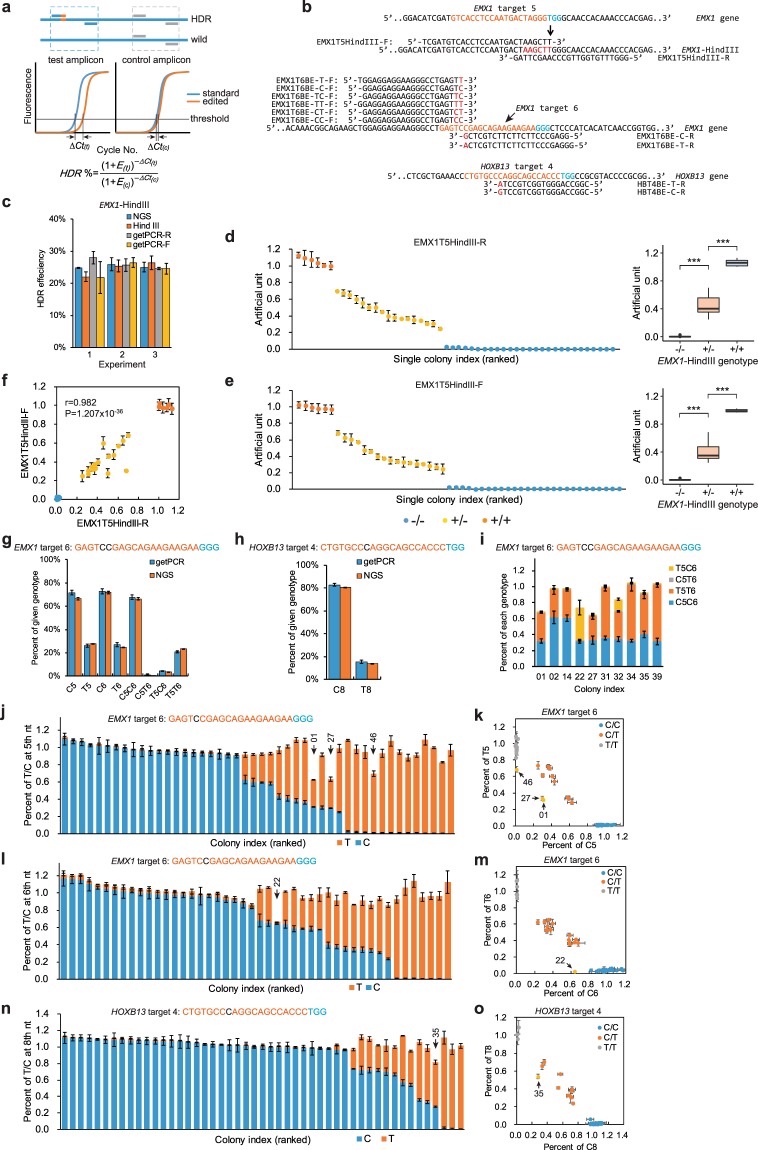
Application of getPCR in HDR and base editing in Lenti-X 293 T cells. (**a**) Schematic overview of the getPCR principle in detection of HDR and base editing. (**b**) Demonstration of getPCR watching primers designed for evaluating HDR efficiency in *EMX1* gene and base editing in *EMX1* and *HOXB13* genes. PAM sequence highlighted in blue, target sequence in orange and watching base in red. (**c**) HDR efficiency quantification on *EMX1* gene target 5 with getPCR in comparison with NGS and HindIII digestion methods. (**d–f**) Single cell clones were isolated and propagated from HDR experiment on *EMX1* gene target 5 and genotyped by getPCR method with two different watching primers alone or in combination. Box plots show quartiles with a band at median, whiskers indicating 1.5 IQR, and outliers shown separately. (**g,h**) Frequency of each genotype determined by getPCR and NGS method in base editing experiment targeting *EMX1* and *HOXB13* gene respectively using watching primes shown in panel b. (**i**) Detailed genotypes of 10 clones from *EMX1* gene base editing experiment which are heterozygous at both 5^th^ and 6^th^ position were further determined by getPCR method using watching primes shown in panel B. (**j,k**) Bar chart and scatterplots display genotyping results of 5^th^ nucleotide of *EMX1* gene target 6 of single-cell clones from base editing experiment. Watching primes for getPCR were shown in panel B. (**l,m**) Single-cell clone genotyping of the 6^th^ nucleotide of *EMX1* gene target 6 in base editing experiment. Watching primes for getPCR were shown in panel B. (**n,o**) Bar chart and scatterplots display of genotyping results of single-cell clones underwent base editing on *HOXB13* gene target 4. Watching primers for getPCR were shown in panel B. (Means ± s.e.m, n = 3 independent technical replicates, *P < 0.05, **P < 0.01, ***P < 0.001).

When it turns to HDR-mediated genome editing, getPCR can determine repair efficiency directly **(**Fig. 6a**)**. Genome editing experiments were performed in Lenti-X 293 T cells with Cas9 and *EMX1* gRNA target 5 together with the HDR template designed to introduce a HindIII site neighbor to PAM sequence **(**Fig. 6b**)**. The getPCR method as well as NGS-based amplicon sequencing and HindIII-mediated restriction fragment length polymorphism (RFLP) analysis were applied to determine the HDR efficiency. Two watching primers designed in forward and reverse direction respectively both could determine the HDR frequencies with comparable level to RFLP and NGS based methods **(**Fig. 6c**)**. The HDR frequencies from three biological samples were evaluated to be around 25%. Furthermore, in genotyping of the 50 single-cell clones derived from this HDR experiment, both two watching primers successfully picked out all the 6 clones homozygous and all the 17 clones heterozygous for HDR event **(**Fig. 6d,e**)**. In addition, detection values with these two watching primers were highly consistent by a strong correlation (r = 0.982, P = 1.207 × 10^−36^) and combination analysis could obviously promote the genotyping especially for heterozygous cell clones **(**Fig. 6f**)**.

### Application of getPCR in base editing detection in cells

We applied getPCR in the base editing experiments with BE4 and gRNA of *EMX1* target 6 or *HOXB13* target 4 in Lenti-X 293 T cells **(**Fig. 6b**)**. For this purpose, watching primers were designed in the same manner to HDR detection. In quantification of base editing frequency, getPCR demonstrated comparable results to NGS-based amplicon sequencing method **(**Fig. 6g,h**)**. For *EMX1* target 6, about 27% ‘C’ bases at the 5^th^ and 6^th^ positions of gRNA targeting sequence were converted into ‘T’. Intriguingly, the base editing at these two positions tended to happen simultaneously and generated T5T6 genotype **(**Fig. 6g**)**. As to base editing with gRNA *HOXB13* target 4, which was designed to terminate the open reading frame early by introducing an in-ahead stop codon ‘TAG’, the C-to-T editing efficiency at the 8^th^ position was around 15%, **(**Fig. 6h**)**.

The Lenti-X 293 T cells that underwent base editing at *EMX1* target 6 or *HOXB13* target 4 were further isolated single-cell clones and subjected to genotyping with getPCR method. For base editing at *EMX1* target 6, 25 out of 46 clones were determined to carry C-to-T conversion at the 5^th^ position **(**Fig. 6j,k**)**, and 22 out of 46 clones were proven to carry C-to-T conversion at the 6^th^ position by getPCR analysis **(**Fig. 6l,m**)**. Intriguingly, clone 01, 27 and 46 might contain extra base other than C and T at 5^th^ position and clone 22 might carry such extra base at 6^th^ position as suggested by the missing percentage of base composition in getPCR detection results **(**Fig. 6j,l**)**. Sanger sequencing of these clones showed that C-to-G base editing happened at the 5^th^ position of clone 01 and 27 and at the 6^th^ position of clone 22 **(**Supplementary Fig. 5a–c**)**. Surprisingly, clone 46 didn’t carry base conversion other than C to T at the 5^th^ nucleotide but had an A-to-T editing at the −8 nucleotide of gRNA targeting sequence on one allele **(**Supplementary Fig. 5c**)**. This A-to-T mutation can be mapped to the 14^th^ nucleotide of primer from the 3′ end and impeded the primer annealing to this allele, which in turn resulted in missed getPCR signal. It is worth noticing that the genotyping values of single cell clones that were heterozygous for genome editing events including indels (Fig. 5c–i, Supplementary Fig. 4a,b), HDR (Fig. 6d,e) as well as base editing (Fig. 6j,l,n) exhibited large variation in comparison to results on plasmid mimics (Fig. 4d–f). Interestingly, the allele percentages of heterozygous clones were usually found to be around 33% or 66% but not 50% in getPCR analysis **(**Fig. 6j,l**)**. Sanger sequencing chromatograms of target genomic region from these clones exhibited highly consistent results **(**Supplementary Figs. 5c, 6b**)**. For example, the percentages of T and C base at the 5^th^ nucleotide position of clone 11 were determined to be 28.8% and 62.9% respectively in getPCR analysis, and consistently the peak height of C base was nearly twice of T in Sanger sequencing. It confirmed that getPCR detection results accurately reflected the real and precise genotypes of single cell clones. On the other hand, the deviation of the allele frequency from 50% may come from two possible resources, the clone might grow from multiple cells or the given region is triploid in the Lenti-X 293 T cells. The latter possibility is preferred and further investigated because the HEK-293 cells, where Lenti-X 293 T came from has been reported to be near triploid with 62–70 chromosomes per cell^38,39^. To address the question, allelic ratio of two heterogenous SNPs, rs6728203 and rs6751051 in the second intron of *EMX1* gene were evaluated by Sanger sequencing in Lenti-X 293 T (Supplementary Fig. 7a). The chromatograms showed that A allele was twice as much as the G allele at rs6728203, and the G allele was twice as much as the A allele at rs6751051. It strongly indicates that *EMX1* gene has three copies. Furthermore, the copy numbers of *HOXB13* and *DYRK1A* genes were then determined using qPCR analysis by taking G allele of rs6728203 as calibration control (Supplementary Fig. 7b). Both *HOXB13* gene and *DYRK1A* gene were proven to have four copies. However, even though Sanger sequencing results available, ten clones were still unknown for allele specific genotype which were heterozygous at both 5^th^ and 6^th^ nucleotide **(**Supplementary Fig. 5c**)**. Four watching primers were designed to further genotyping these clones through getPCR method **(**Fig. 6b**)**, and the exact allele-specific genotypes of these clones were successfully determined **(**Fig. 6i**)**. Clone 02 and 14 were defined to be C5C6/C5C6/T5T6, and clone 31, 34, 35 as well as 39 were proven to be C5C6/T5T6/T5T6. Clone 01 and 27 were found both to be C5C6/T5T6/G5C6 and clone 22, 32 were finally determined to be C5C6/T5C6/T5G6 and C5C6/T5T6/T5C6 respectively.

For base editing at *HOXB13* target 4 to introduce an in-frame stop codon, 14 out of 49 clones were determined to carry C-to-T conversion at the 8^th^ position **(**Fig. 6n–o**)**. Notably, clone 35 could possibly carry extra base other than C and T bases at this position as suggested by missing part in base percentage in getPCR detection. Sanger sequencing chromatograms showed that a C-to-G base editing happened at the 8^th^ position on one of the three alleles **(**Supplementary Fig. 6a,b**)**. Similarly, getPCR could also determine the delicate genotypes of heterozygous clones as verified by sanger sequencing. For example, six clones 13, 45, 42, 16, 02 and 33 were genotyped to be C/C/T at the 8^th^ nucleotide of *HOXB13* gRNA target 4 sequence.

## Discussion

As the rapid development and wide application of CRISPR technology, a simple but accurate and reliable method that can determine the editing efficiency is in great demand for prescreening to choose the highly effective gRNA targets or experiment strategies, as well as for single-cell clone genotyping. A good method for these purposes is supposed to be simple in experiment procedure, reliable in quantification result, time-saving and low cost as well as not requiring specific devices that not readily available in major laboratories. The getPCR method that requires only one qPCR step has been fully proven to meet all these requirements, especially to be the most simple, fast and less costly one. With the simulated indels at *HOXB13* target 1, getPCR could precisely determine the indel frequencies at full range from 0% to 100%, whereas the values from Surveyor method exhibited huge deviation from the real values when indel frequencies were higher than 40%. The less accuracy of Surveyor method is mainly caused by self-annealing between the same indels as well as incomplete digestion of mismatched strands. On the other hand, the +1 insertion/−1 deletion pattern occupying the most of mutation forms in the Cas9 induced NHEJ can be efficiently recognized by getPCR. Taken the 3-watching-bases primer for example, the +1 insertion will actually cause slipping of 3 but not 1 watching bases at primer 3′ end, and the −1 deletion will cause slipping of 3 or 4 watching bases at primer 3′ end depending on the deletion position. This will endow getPCR method great sensitivity to almost all forms of indels. For *in vivo* genome editing in cells, getPCR accurately determined indel frequencies at all nine CRISPR targets with comparable results to NGS based methods except not providing detailed sequence information. In addition, getPCR also quantified HDR efficiency at *EMX1* target 5 and base editing efficiency at *EMX1* target 6 and *HOXB13* target 4 accurately on a par with NGS method in all cases. Of note, CRISPR Cas9 exhibited extremely diverse editing efficiency levels from 6% to more than 80% at the nine targets. Find the factors that determine the editing efficiency level of CRISPR targets, elucidate the underlying mechanism and subsequently develop a tool to increase editing efficiency will improve the development and application of genome editing technology.

Compared to PCR based methods previously reported by Yu *et al*.^33^ and Hua *et al*.^34^, our method possesses several improvements. Firstly, getPCR method employs real time PCR technology that talented for nucleic acid quantification while the present two methods mainly base on common PCR. Secondly, for the crucial watching primer design step, Yu’s work didn’t describe primer design principle, and Hua’s work suggested 4 watching bases only based on speculation. We defined 3, 4 and 5 watching bases as the watching primer design roles for their optional and comparable ability in discriminating indels based on solid experimental results from systematic investigation. This provides researchers more options in designing primers and hence makes this method more practical. Thirdly, the present two methods can detect indels only, while getPCR method also possesses the ability to detect HDR and base editing events. Fourthly, high-fidelity KOD FX DNA polymerase was used in Hua’s work in determining the critical annealing temperature and indel detection for three genes^34^. However, our works indicate that high-fidelity DNA polymerases are not applicable in watching primer-based methods because they can remove mismatch nucleotide by its 3′ to 5′ exonuclease activity and destroy the indel discrimination ability of watching primers.

For application in efficiency determination of HDR and base editing performed in human genome, the sensitivity is estimated to be around 0.1% if 10 ng genomic DNA loaded as template in getPCR analysis. In detecting indel frequency lower than 10% the confidence level is unfavorable because the indel efficiency is calculated indirectly from wild type percent in the mixture. Nevertheless, the sensitivity of indel frequency detection can be further improved by raising the number of repeating wells or technical replications in getPCR. Notably, genome editing efficiency evaluation is mainly demanded in prescreening of gRNA candidates to choose those highly active for subsequent experiments. Usually CRISPR targets with higher editing efficiencies, such like more than 50% are specially concerned and tend to be chosen for further applications, whereas less active gRNAs with editing efficiencies lower than 10% are on the contrary seldom desired. In such situation, the less ideal performance of getPCR in quantifying low level indel frequency will not actually raise problem in majority applications.

When applied to single-cell clone genotyping for desired genome modification, getPCR method correctly determined the genotypes of all clones as verified by Sanger sequencing in all cases including NHEJ mediated indels, HDR mediated modification as well as base editing generated by BE4. Notably, getPCR displayed great competence in telling the exact allele number that carrying anticipated modifications. Even more surprisingly, getPCR method was able to define the ploidy characters at the concerned genomic regions in Lenti-X 293 T cells. It provided strong supports to expand getPCR method into other application fields such like moderate-to-high throughput SNP genotyping and detecting chromosome abnormalities such like trisomy in clinical diagnosis of chromosomal disorders.

This getPCR technology has several advantages over present methods. Firstly, the getPCR method uses genomic DNA as real-time PCR template directly, without involving a pre-amplification PCR step like other methods including Surveyor/T7E1 method, qEva-CRISPR, IDAA as well as NGS-based amplicon sequencing. It makes getPCR free from potential bias introduced during PCR amplification, which might impair the precision of indel frequency detection. As widely known, large deletions that failing pre-amplification will be overlooked in the quantification process and result in underestimated editing efficiency value. This possibility might explain very well our observations that getPCR detection values are often slightly higher than the NGS based methods in indel frequency determination. Secondly, compared to Surveyor/T7E1 and HRM methods, getPCR technology is less likely to be affected by SNPs neighboring the editing site in the PCR amplicon region. Thirdly, getPCR utilizes the power of real-time PCR in relative nucleic acid quantification with un-edited genomic DNA sample as 100% wild standard to realize the determination of indel frequency. This endows getPCR to be the most reliable method in comparison to other optical density-based ones such as Surveyor/T7E1, RFLP analysis, ENIT and SSCP^40^, or fluorescence strength-based methods including TIDE^11^ and IDAA. Fourthly, the strategies employed in getPCR to improve the competence in discriminating modified genomic DNA can be further applied in ddPCR based method to increase its accuracy. Finally, because only involving one real-time PCR step, getPCR is supposed to be the fastest, most reliable and less costly one for editing efficiency quantification and single-cell clone genotyping.

On the other hand, getPCR also have limitations. As a method based on traditional real-time PCR, it can mainly be used in the characterization of genome modifications at on-target and some predictive off-target site of engineered nuclease. It can’t be used for investigation of genome-wide off-target cutting profiles, which can be achieved by other methods including BLESS^41^, GUIDE-seq.^42^ and dCas9-based ChIP-seq.^43^ etc. Meantime, getPCR method can provide only genome editing efficiency result but not detailed sequence information like NGS method does. In addition, application of getPCR method is supposed to be constrained for gRNA targets in highly repetitive sequence regions and high GC content regions that hard to design PCR primers. Finally, even though not encountered in our experiments, in certain rare cases, potential indel might not result in primer-template mismatch and be overlooked in the detection if single watching primer employed only. However, this problem can be eliminated by combining two rounds of detection using forward and reverse watching primers respectively.

Overall, getPCR provides a common way to evaluate the genome modifications generated by RNA guided nucleases. It can be easily further extended for use in genome editing evaluation of other nucleases which have predictable cutting position, including zinc finger nuclease (ZFNs)^44^, transcription activator-like effector nucleases (TALENs)^45^ and CRISPR RNA-guided FokI nuclease (RFNs)^46,47^, paired Cas9 nickases^48,49^ in any kind of cells that capable of NHEJ repair in response to double-strand DNA break. Undoubtedly the application of getPCR in detecting genome editing generated by other nucleases will demand further investigations for the rules in designing watching primers. The strategies described here can be used for routine editing efficiency evaluation in pre-screening of gRNAs before formal experiments and for single-cell clone genotyping. This method will hopefully further boom the wide application of genome editing technologies in molecular and cellular biology researches in the future.

## Materials and Methods

### Plasmids and oligos

The plasmid containing *HOXB13* gene coding region in pcDNA3.1 vector was gifted by professor GH Wei from University of Oulu^50^. 26 DNA variants simulating different potential indels at *HOXB13* gRNA target 1 (Fig. 2a) and other 15 variants containing mutations to introduce different types of primer-template mismatches were constructed through site-directed mutagenesis. The sgRNA expression plasmid was constructed by deleting Cas9 expression cassette from pSpCas9(BB) vector (Addgene, #42230)^2^ through PCR method. To construct plasmids expressing sgRNAs, annealed oligo pairs bearing 20-nt guide sequences were ligated into the sgRNA expression plasmid or the pSpCas9(BB) vector between BbsI sites. The high-fidelity CRISPR-Cas9 nuclease(R661A/Q695A/Q926A)^37^ used in our whole work was obtained through site-directed mutagenesis on the basis of pSpCas9(BB). BE4-Gam plasmid (Addgene, #100806)^51^ was used for base editing experiments. The 99-nt single strand HDR template containing *EMX1*-HindIII mutation neighbor to the PAM sequence of *EMX1* gRNA target 5 were synthetized in Invitrogen Trading (Shanghai) Co. Ltd. The *EMX1* gene containing HindIII variation was also cloned into a plasmid and used in HDR efficiency calculation. Sequences of all the used primers and oligos are shown in Supplementary Table 1.

### Cell culture

The Lenti-X 293 T cells was originally purchased from Clontech Laboratories Inc. (Cat#632180) and cultured in Dulbecco’s Modified Eagle’s Medium (Gibco, Cat#C11995500BT) supplemented with 1×penicillin/streptomycin (HyClone, Cat#SV30010) and 10% (v/v) FBS (Gibco, Cat#10270-106), at 37 °C with 5% CO_2_. It was checked regularly for mycoplasma using MycoBlue^TM^ Mycoplasma Detector kit according to product manual (Vazyme, Cat#D101-01). The cell line was proven to be mycoplasma free during our study.

### Transfections

The Lenti-X 293 T cells were seeded into 24-well plates (Labserv, Cat#310109007) at a density of 120,000 cells per well the day before transfection. Cells were transfected at ~70% confluency using Lipofectamine 2000 (ThermoFisher Scientific, Cat#11668019) according to the manufacturer’s instruction. For indel detection, 1 μg of plasmid that expressing both sgRNA and high-fidelity CRISPR-Cas9 was applied in each transfection. For base editing, 750 ng of BE4 plasmid and 250 ng of sgRNA expression plasmid were used for each transfection. For HDR-mediated genome modification, 600 ng of plasmid that expressing both sgRNA and high-fidelity CRISPR-Cas9 as well as 10pmol HDR oligo were used for each transfection. 48 h after transfection, genomic DNA was extracted with a TIANamp Genomic DNA Kit (TIANGEN, Cat#DP304-03) according to the manufacturer’s instruction.

### getPCR conditions

For each getPCR reaction, 0.1 ng of plasmid DNA or 2.5 ng of genomic DNA was used as template in 15 μl reaction system of AceQ qPCR SYBR Green Master Mix (Vazyme, Cat#Q111-02). Real-time PCR was run on the thermocyclers Rotor-Gene Q (Qiagen, Germany) using the following program: initial denaturation at 95 °C for 5 min, then 40 cycles at 95 °C for 30 s, 65–69 °C for 30 s and at 72 °C for 10 s with fluorescence acquirement, followed by a standard melting curve step. While employing LightCycler® 96 thermal cycler Instrument (Roche Applied Science, Germany), the following conditions were used: initial denaturation at 95 °C for 5 min, then 40cycles at 95 °C for 15 s, at 65–69 °C for 20 s and at 72 °C for 10 s, with fluorescence acquirement, followed by a standard melting curve stage. The primer Tm value is calculated using the online Oligo Calc tool^52^.

### Indel frequency quantification using getPCR

The 26 plasmids mimicking different type of indels were mixed equally and regarded as 100% indels (Fig. 2a), which can be mixed further with wild type DNA at given ratio to obtain DNA samples with diverse indel frequencies. The indel frequencies were evaluated using getPCR method. In getPCR assay, 0.1 ng of plasmid DNA was used as template for each qPCR reaction. The wild type percentage in the mixture sample and indel frequency were calculated as described in Fig. 1b. Simultaneously, each of these 26 plasmids was used to simulate single-cell clones with homozygous indel. Each plasmid was also equally mixed with wild type DNA plasmid to simulate heterozygous single-cell clones that bearing indel on one allele. Sequences of the getPCR primers are shown in Supplementary Table 2. As to indel frequency quantification on genomic DNA sample, 2.5 ng of genomic DNA was included as template and amplified using primer as summarized in Supplementary Table 3.

### Surveyor nuclease assay

Indel frequencies were also determined using surveyor nuclease assay method with Surveyor® Mutation Detection Kits (Integrated DNA Technologies, Cat#706020) as described previously^3^. In brief, genomic DNA was extracted using TIANamp Genomic DNA Kit (TIANGEN, Cat#DP304-03) according to product manual. DNA regions were then amplified with the cut site 200–400 bp away from each end using high-fidelity PrimeSTAR® Max DNA Polymerase (TaKaRa, Cat#R045B) and primers summarized in Supplementary Table 2a. 270 ng of purified PCR product was subjected to heteroduplex formation using a T100™ Thermal Cycler (Bio-Rad) and subsequently treated with Surveyor Nuclease according to user guide. The DNA fragments were separated on 2% agarose gel and images were acquired using Quantum-ST5 (VILBER LOURMAT, France) and analyzed with Quantum ST5 Xpress software.

### Application of getPCR in HDR and BE4 experiments

Variation-specific getPCR primers were designed with modified nucleotide(s) at 3′ end as summarized in Supplementary Table 3. In getPCR analysis, 2.5 ng of genomic DNA was included as template for each reaction. The genome modification efficiencies were calculated using the equation as shown in Fig. 6a.

### HindIII-based RFLP assay

In the HDR experiments targeting *EMX1* gene, one HindIII site was introduced neighbor to the PAM sequence, which enabled HDR efficiency quantification through HindIII-based restriction fragment length polymorphism (RFLP) analysis. Briefly, 639 bp of DNA region with HindIII site 355 bp away from 5′ end was amplified using PrimeSTAR® Max DNA Polymerase and primers same to Surveyor assay as shown in Supplementary Table 2a and purified using Universal DNA Purification Kit (TIANGEN, Cat#DP214). 270 ng of PCR product was subjected to HindIII digestion and resolved on a 2% agarose gel. The images were acquired using Quantum-ST5 (VILBER LOURMAT, France) and analyzed with Quantum ST5 Xpress software.

### NGS-based methods

DNA regions covering genome modification were amplified to construct NGS libraries and editing efficiencies were then calculated by counting the NGS reads. Sequencing libraries were prepared with two rounds of PCR amplifications with genomic DNA as template. In the first round PCR, amplicons of 250–280 bp were designed with the Cas9 cutting site near the middle part and the binding sites of Illumina sequencing primers were introduced at both ends. In the second round PCR, adaptors for cluster generation and index sequences were attached. After purification and quantification, the libraries were subjected to 150 bp paired-end sequencing on the Illumina HiSeq X-TEN platform run by Genewiz. For NHEJ mediated indels, the wild type read counts in each library were acquired with wild type DNA sequence and the indel editing efficiency was calculated using the equation “Editing efficiency = 1-wide_type_counts/total_counts*100%”. As to modification efficiency in base editing and HDR experiments, the read counts of expected DNA variation sequences in the library were acquired and editing efficiencies were calculated using the equation “Efficiency = expected_sequence_counts/total_counts*100%”. Full details of the library preparation and counting method can be found in Supplementary Table 4.

### Single cell cloning and genotyping

About 48 hours post transfection, single cells were isolated by limited dilution method and grown in 96-well plates. When reached confluent, cells were further propagated into 24-well plates and grew until confluent. Genomic DNA from single-cell clones was isolated with a TIANamp Genomic DNA Kit (TIANGEN, Cat#DP304–03) according to the manufacturer’s instructions. The genotype of each clone was determined by getPCR assay and confirmed by Sanger sequencing of amplicon covering the cutting site. PCR amplifications were performed with high-fidelity PrimeSTAR® Max DNA Polymerase (TaKaRa, Cat#R045B) and primers as shown in Supplementary Table 2a. PCR products were then subjected to Sanger sequencing (TsingKe Biological Technology or GeneWiz). To determine the exact sequence of each allele for heterozygous cells, the Sanger sequencing ab1 files were directly analyzed with TIDE Web Tool (https://tide.nki.nl/)^11^. Alternatively, the amplicons were further cloned into vector and single cell clones were analyzed by Sanger sequencing. To determine the gene copy number of *HOXB13*, *EMX1* and *DYRK1A* in Lenti-X 293 T, the G allele of rs6728203, a SNP locating in the second intron of *EMX1* gene was used as reference for calculation in qPCR analysis. This SNP is heterozygous in Lenti-X 293 T cells and contains one G allele and two A alleles as shown in Sanger sequencing. In an author blood DNA, it is diploid and contains one G allele and one A allele. Primers were listed in Supplementary Table 5.

### Sensitivity of different DNA polymerases to mismatch

A variety of commercial DNA polymerase products were evaluated for their sensitivity to primer mismatch. They are 2×Taq master mix (Vazyme, Cat#P111, Lot#511151), Premix Taq™ (TaKaRa, Cat#RR901, Lot#A3001A), NOVA Taq-Plus PCR Forest Mix (Yugong Biolabs, Cat#EG15139, Lot#1393216101), DreamTaq Green PCR Master Mix (ThermoFisher, Cat#K1081, Lot#00291017), Platinum™ Green Hot Start PCR Master Mix (Invitrogen, Cat#13001012, Lot#00401653), PrimeSTAR® Max DNA Polymerase (TaKaRa, Cat#R045, Lot#AI51995A), Phusion Hot Start II high-Fidelity PCR Master Mix (ThermoFisher, Cat#F-565, Lot#00633307) as well as Q5® Hot Start high-Fidelity DNA Polymerase (NEB, Cat#M0493). In a 20 μl reaction system, 10 ng of plasmid DNA was included as template and thermal cycled with the programs as suggested by given product manuals. PCR products were then subjected to 2.0% agarose gel electrophoresis and Sanger sequencing directly. Gel images were acquired using Quantum-ST5 (VILBER LOURMAT, France) and analyzed with Quantum ST5 Xpress software.

### Comparison of different qPCR SYBR green products in getPCR

To test the extensive usability of getPCR, multiple qPCR SYBR mix products were investigated including AceQ qPCR SYBR Green Master Mix (Vazyme, Cat#Q111-02), SYBR™ Select Master Mix (Applied Biosystems™, Cat#4472908), Power SYBR Green PCR Master Mix (Applied Biosystems™, Cat#4367659), QuantiNova SYBR Green PCR Kit (QIAGEN, Cat#208054), FastStart Essential DNA Green Master (Roche, Cat#06402712001), NovoScript® SYBR One-Step qRT-PCR SuperMix (novoprotein, Cat#E092-01A), 2 × T5 Fast qPCR Mix (TSINGKE, Cat#TSE202), UltraSYBR Mixture (CWBIO, Cat#CW0957), SYBR Premix Ex Taq (TaKaRa, Cat#RR420, A5405-1). Real-time qPCRs were run on the thermocyclers Rotor-Gene Q (Qiagen, Germany) or LightCycler® 96 thermal cycler Instrument (Roche Applied Science, Germany). The PCR and qPCR conditions were set according to the manufacturer’s protocol with given annealing temperature.

### Statistical analysis

Student’s t tests (two-tailed) were applied based on the results of Levene test to assess the statistical significance of getPCR results for single-cell clone genotyping using IBM SPSS Statistics version 21. The correlation between two different getPCR strategies were assessed with Pearson test using IBM SPSS Statistics version 21 software.

## Supplementary information

Supplementary information

## Data Availability

All datasets generated during the current study are available from the corresponding author upon reasonable request.
