# Supplementary material for: A qPCR method for genome editing efficiency determination and single-cell clone screening in human cells
Source: Sci Rep. 2019 Dec 11;9:18877. doi: 10.1038/s41598-019-55463-6 (PMC6906436; doi:10.1038/s41598-019-55463-6)
Supplement: Supplementary file 1 — Supplementary information [file 41598_2019_55463_MOESM1_ESM.pdf]

A qPCR method for genome editing efficiency determination and single-cell clone screening in human cells

Bo Li[1], Naixia Ren[1], Lele Yang[1], Junhao Liu[1], Qilai Huang[1,2,3]

1 Shandong Provincial Key Laboratory of Animal Cell and Developmental Biology, School of Life Sciences, Shandong University, Qingdao, China;

2 State Key Laboratory of Microbial Technology, Shandong University, Qingdao, China

3 The Second Hospital of Shandong University, Jinan, China

Correspondence should be addressed to Q.H. (qlhuang@sdu.edu.cn)

Fig. S1

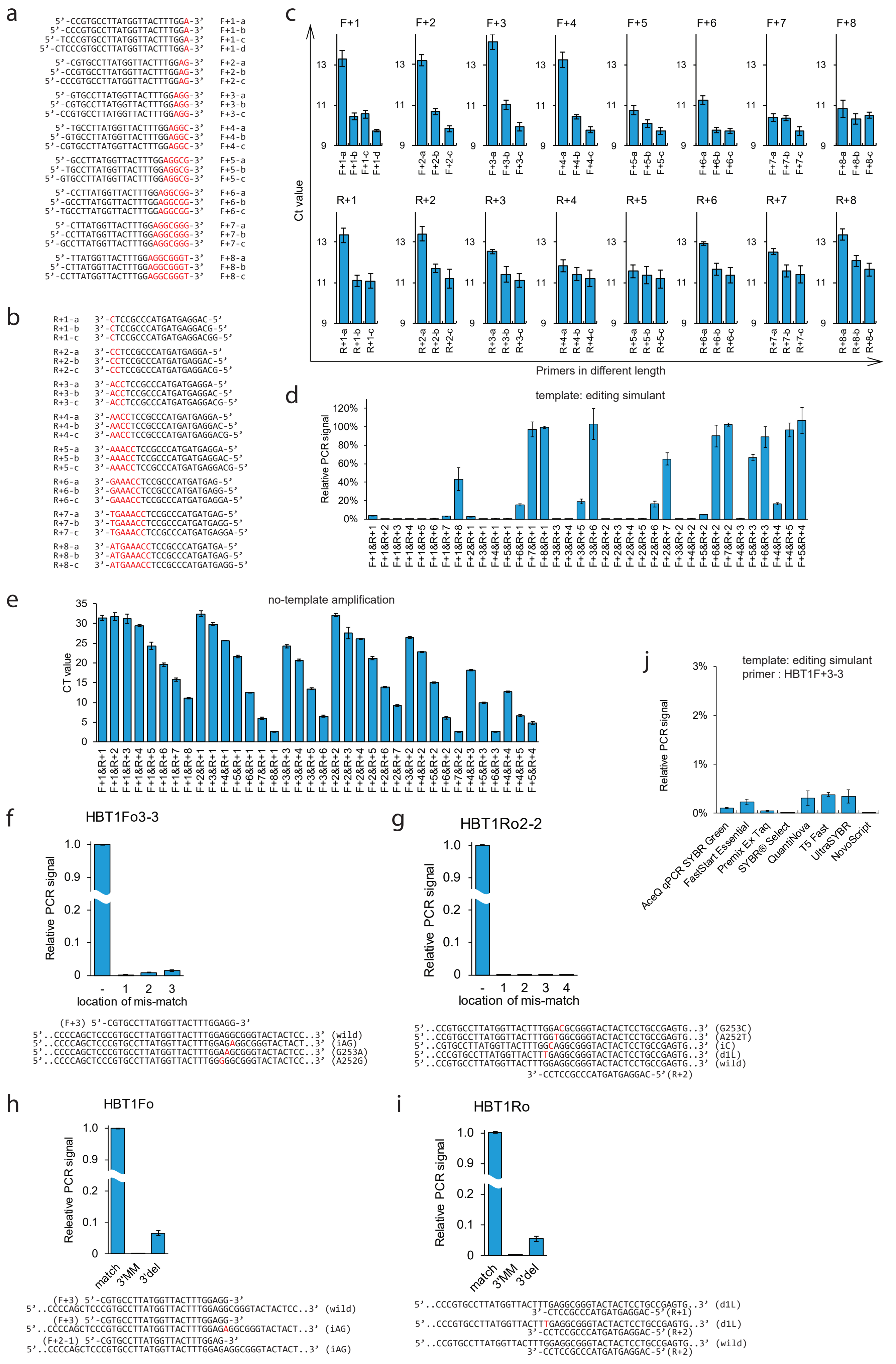
a
5’-CCGTGCCTTATGGTTACTTTGGA-3’ F+1-a
5’-CCCGTGCCTTATGGTTACTTTGGA-3’ F+1-b
5’-TCCCGTGCCTTATGGTTACTTTGGA-3’ F+1-c
5’-CTCCCGTGCCTTATGGTTACTTTGGA-3’ F+1-d
5’-CGTGCCTTATGGTTACTTTGGAG-3’ F+2-a
5’-CCGTGCCTTATGGTTACTTTGGAG-3’ F+2-b
5’-CCCGTGCCTTATGGTTACTTTGGAG-3’ F+2-c
5’-GTGCCTTATGGTTACTTTGGAGG-3’ F+3-a
5’-CGTGCCTTATGGTTACTTTGGAGG-3’ F+3-b
5’-CCGTGCCTTATGGTTACTTTGGAGG-3’ F+3-c
5’-TGCCTTATGGTTACTTTGGAGGC-3’ F+4-a
5’-GTGCCTTATGGTTACTTTGGAGGC-3’ F+4-b
5’-CGTGCCTTATGGTTACTTTGGAGGC-3’ F+4-c
5’-GCCTTATGGTTACTTTGGAGGCG-3’ F+5-a
5’-TGCCTTATGGTTACTTTGGAGGCG-3’ F+5-b
5’-GTGCCTTATGGTTACTTTGGAGGCG-3’ F+5-c
5’-CCTTATGGTTACTTTGGAGGCGG-3’ F+6-a
5’-GCCTTATGGTTACTTTGGAGGCGG-3’ F+6-b
5’-TGCCTTATGGTTACTTTGGAGGCGG-3’ F+6-c
5’-CTTATGGTTACTTTGGAGGCGGG-3’ F+7-a
5’-CCTTATGGTTACTTTGGAGGCGGG-3’ F+7-b
5’-GCCTTATGGTTACTTTGGAGGCGGG-3’ F+7-c
5’-TTATGGTTACTTTGGAGGCGGGT-3’ F+8-a
5’-CTTATGGTTACTTTGGAGGCGGGT-3’ F+8-b
5’-CCTTATGGTTACTTTGGAGGCGGGT-3’ F+8-c
b
R+1-a 3’-CTCCGCCCATGATGAGGAC-5’
R+1-b 3’-CTCCGCCCATGATGAGGACG-5’
R+1-c 3’-CTCCGCCCATGATGAGGACGG-5’
R+2-a 3’-CCTCCGCCCATGATGAGGA-5’
R+2-b 3’-CCTCCGCCCATGATGAGGAC-5’
R+2-c 3’-CCTCCGCCCATGATGAGGACG-5’
R+3-a 3’-ACCTCCGCCCATGATGAGGA-5’
R+3-b 3’-ACCTCCGCCCATGATGAGGAC-5’
R+3-c 3’-ACCTCCGCCCATGATGAGGACG-5’
R+4-a 3’-AACCTCCGCCCATGATGAGGA-5’
R+4-b 3’-AACCTCCGCCCATGATGAGGAC-5’
R+4-c 3’-AACCTCCGCCCATGATGAGGACG-5’
R+5-a 3’-AAACCTCCGCCCATGATGAGGA-5’
R+5-b 3’-AAACCTCCGCCCATGATGAGGAC-5’
R+5-c 3’-AAACCTCCGCCCATGATGAGGACG-5’
R+6-a 3’-GAAACCTCCGCCCATGATGAG-5’
R+6-b 3’-GAAACCTCCGCCCATGATGAGG-5’
R+6-c 3’-GAAACCTCCGCCCATGATGAGGA-5’
R+7-a 3’-TGAAACCTCCGCCCATGATGAG-5’
R+7-b 3’-TGAAACCTCCGCCCATGATGAGG-5’
R+7-c 3’-TGAAACCTCCGCCCATGATGAGGA-5’
R+8-a 3’-ATGAAACCTCCGCCCATGATGA-5’
R+8-b 3’-ATGAAACCTCCGCCCATGATGAG-5’
R+8-c 3’-ATGAAACCTCCGCCCATGATGAGG-5’
c
F+1 F+2 F+3 F+4 F+5 F+6 F+7 F+8
R+1 R+2 R+3 R+4 R+5 R+6 R+7 R+8
Ct value
Primers in different length
d
template: editing simulant
Relative PCR signal
e
no-template amplification
CT value
f
HBT1Fo3-3
Relative PCR signal
location of mis-match
(F+3) 5’-CGTGCCTTATGGTTACTTTGGAGG-3’
5’..CCCCAGCTCCCGTGCCTTATGGTTACTTTGGAGGCGGGTACTACTCC..3’ (wild)
5’..CCCCAGCTCCCGTGCCTTATGGTTACTTTGGAGAGGCGGGTACTACT..3’ (iAG)
5’..CCCCAGCTCCCGTGCCTTATGGTTACTTTGGAAGCGGGTACTACTCC..3’ (G253A)
5’..CCCCAGCTCCCGTGCCTTATGGTTACTTTGGGGGCGGGTACTACTCC..3’ (A252G)
g
HBT1Ro2-2
Relative PCR signal
location of mis-match
5’..CCGTGCCTTATGGTTACTTTGGACGCGGGTACTACTCCTGCCGAGTG..3’ (G253C)
5’..CCGTGCCTTATGGTTACTTTGGTGGCGGGTACTACTCCTGCCGAGTG..3’ (A252T)
5’..CGTGCCTTATGGTTACTTTGGCAGGCGGGTACTACTCCTGCCGAGTG..3’ (iC)
5’..CCCGTGCCTTATGGTTACTTTGAGGCGGGTACTACTCCTGCCGAGTG..3’ (d1L)
5’..CCGTGCCTTATGGTTACTTTGGAGGCGGGTACTACTCCTGCCGAGTG..3’ (wild)
3’-CCTCCGCCCATGATGAGGAC-5’(R+2)
h
HBT1Fo
Releative PCR signal
match 3’MM 3’del
(F+3) 5’-CGTGCCTTATGGTTACTTTGGAGG-3’
5’..CCCCAGCTCCCGTGCCTTATGGTTACTTTGGAGGCGGGTACTACTCC..3’ (wild)
(F+3) 5’-CGTGCCTTATGGTTACTTTGGAGG-3’
5’..CCCCAGCTCCCGTGCCTTATGGTTACTTTGGAGAGGCGGGTACTACT..3’ (iAG)
(F+2-1) 5’-CGTGCCTTATGGTTACTTTGGAG-3’
5’..CCCCAGCTCCCGTGCCTTATGGTTACTTTGGAGAGGCGGGTACTACT..3’ (iAG)
i
HBT1Ro
Relative PCR signal
match 3’MM 3’del
5’..CCCGTGCCTTATGGTTACTTTGAGGCGGGTACTACTCCTGCCGAGTG..3’ (d1L)
3’-CTCCGCCCATGATGAGGAC-5’(R+1)
5’..CCCGTGCCTTATGGTTACTTTGAGGCGGGTACTACTCCTGCCGAGTG..3’ (d1L)
3’-CCTCCGCCCATGATGAGGAC-5’(R+2)
5’..CCGTGCCTTATGGTTACTTTGGAGGCGGGTACTACTCCTGCCGAGTG..3’ (wild)
3’-CCTCCGCCCATGATGAGGAC-5’(R+2)
j
template: editing simulant
primer : HBT1F+3-3
Relative PCR signal
AceQ qPCR SYBR Green
FastStart Essential
Premix Ex Taq
SYBR® Select
QuantiNova
T5 Fast
UltraSYBR
NovoScript

Supplementary Figure 1 Considerations in designing getPCR primers and running getPCR. (**a, b**) Design of multiple getPCR primers with given watching bases but different length/Tm value, in forward and reverse direction respectively. (**c**) Amplification efficiency of these getPCR primers on wild type template. (**d**) Bar chart showing PCR specificity of watching primer combinations with indel mimic plasmids as template, alternative exhibition of **Figure 2e**. (**e**) Bar chart showing PCR self-amplification signal of watching primer combinations without adding template, alternative exhibition of **Figure 2f**. (**f, g**) Influence of single-base mismatch position relative to 3' end on the PCR amplification, forward and reverse watching primer respectively. (**h, i**) Comparison of 3' end base mismatch with 3' end base deletion for their ability in hampering PCR amplification, forward and reverse watching primer respectively. (**j**) Comparison of multiple qPCR SYBR green mix products for their suitability in getPCR application. (Means ± s.e.m, n=3 independent technical replicates)

Fig. S2

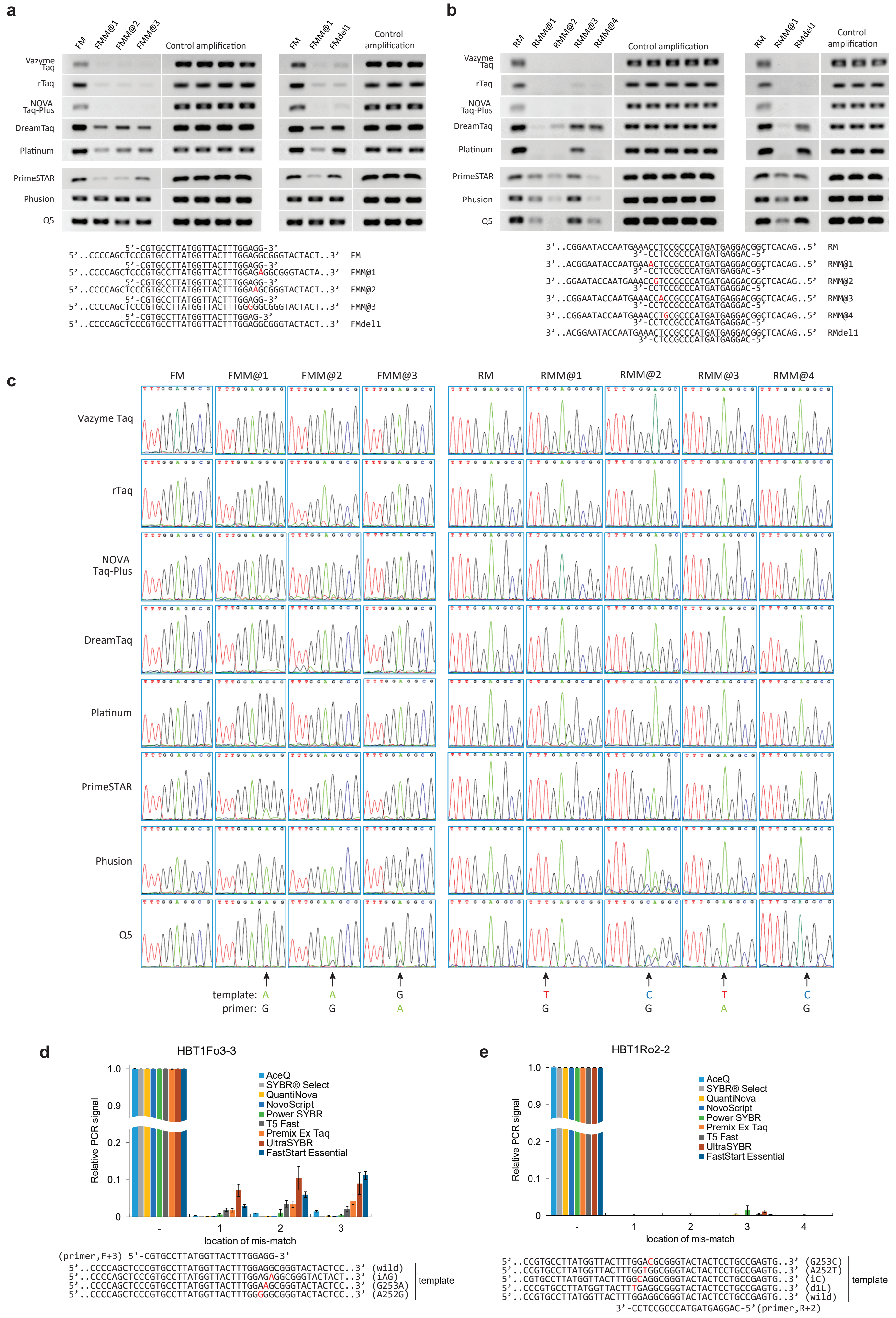
a
FM
FMM@1
FMM@2
FMM@3
Control amplification
FM
FMM@1
FMdel1
Control amplification
Vazyme Taq
rTaq
NOVA Taq-Plus
DreamTaq
Platinum
PrimeSTAR
Phusion
Q5
5'-CGTGCCTTATGGTTACTTTGGAGG-3'
5'..CCCCAGCTCCCGTGCCTTATGGTTACTTTGGAGGCGGGTACTACT..3' FM
5'-CGTGCCTTATGGTTACTTTGGAGG-3'
5'..CCCCAGCTCCCGTGCCTTATGGTTACTTTGGAGAGGCGGGTACTA..3' FMM@1
5'-CGTGCCTTATGGTTACTTTGGAGG-3'
5'..CCCCAGCTCCCGTGCCTTATGGTTACTTTGGAAGCGGGTACTACT..3' FMM@2
5'-CGTGCCTTATGGTTACTTTGGAGG-3'
5'..CCCCAGCTCCCGTGCCTTATGGTTACTTTGGGGGCGGGTACTACT..3' FMM@3
5'-CGTGCCTTATGGTTACTTTGGAG-3'
5'..CCCCAGCTCCCGTGCCTTATGGTTACTTTGGAGGCGGGTACTACT..3' FMdel1
b
RM
RMM@1
RMM@2
RMM@3
RMM@4
Control amplification
RM
RMM@1
RMdel1
Control amplification
Vazyme Taq
rTaq
NOVA Taq-Plus
DreamTaq
Platinum
PrimeSTAR
Phusion
Q5
3'..CGGAATACCAATGAAACCTCCGCCCATGATGAGGACGGCTCACAG..5' RM
3'-CCTCCGCCCATGATGAGGAC-5'
3'..ACGGAATACCAATGAAACTCCGCCCATGATGAGGACGGCTCACAG..5' RMM@1
3'-CCTCCGCCCATGATGAGGAC-5'
3'..GGAATACCAATGAAACCGTCCGCCCATGATGAGGACGGCTCACAG..5' RMM@2
3'-CCTCCGCCCATGATGAGGAC-5'
3'..CGGAATACCAATGAAACCACCGCCCATGATGAGGACGGCTCACAG..5' RMM@3
3'-CCTCCGCCCATGATGAGGAC-5'
3'..CGGAATACCAATGAAACCTGCGCCCATGATGAGGACGGCTCACAG..5' RMM@4
3'-CCTCCGCCCATGATGAGGAC-5'
3'..ACGGAATACCAATGAAACTCCGCCCATGATGAGGACGGCTCACAG..5' RMdel1
3'-CTCCGCCCATGATGAGGAC-5'
c
FM
FMM@1
FMM@2
FMM@3
RM
RMM@1
RMM@2
RMM@3
RMM@4
Vazyme Taq
rTaq
NOVA Taq-Plus
DreamTaq
Platinum
PrimeSTAR
Phusion
Q5
template: A A G T C T C
primer: G G A G G A G
d
HBT1Fo3-3
Relative PCR signal
1.0
0.9
0.2
0.1
0
AceQ
SYBR® Select
QuantiNova
NovoScript
Power SYBR
T5 Fast
Premix Ex Taq
UltraSYBR
FastStart Essential
-
1
2
3
location of mis-match
(primer,F+3) 5'-CGTGCCTTATGGTTACTTTGGAGG-3'
5'..CCCCAGCTCCCGTGCCTTATGGTTACTTTGGAGGCGGGTACTACTCC..3' (wild)
5'..CCCCAGCTCCCGTGCCTTATGGTTACTTTGGAGAGGCGGGTACTACT..3' (iAG)
5'..CCCCAGCTCCCGTGCCTTATGGTTACTTTGGAAGCGGGTACTACTCC..3' (G253A)
5'..CCCCAGCTCCCGTGCCTTATGGTTACTTTGGGGGCGGGTACTACTCC..3' (A252G)
template
e
HBT1Ro2-2
Relative PCR signal
1.0
0.9
0.2
0.1
0
AceQ
SYBR® Select
QuantiNova
NovoScript
Power SYBR
Premix Ex Taq
T5 Fast
UltraSYBR
FastStart Essential
-
1
2
3
4
location of mis-match
5'..CCGTGCCTTATGGTTACTTTGGACGCGGGTACTACTCCTGCCGAGTG..3' (G253C)
5'..CCGTGCCTTATGGTTACTTTGGTGGCGGGTACTACTCCTGCCGAGTG..3' (A252T)
5'..CGTGCCTTATGGTTACTTTGGCAGGCGGGTACTACTCCTGCCGAGTG..3' (iC)
5'..CCCGTGCCTTATGGTTACTTTGAGGCGGGTACTACTCCTGCCGAGTG..3' (d1L)
5'..CCGTGCCTTATGGTTACTTTGGAGGCGGGTACTACTCCTGCCGAGTG..3' (wild)
3'-CCTCCGCCCATGATGAGGAC-5'(primer,R+2)
template

Supplementary Figure 2 Performance of different DNA polymerase products in mismatch discrimination. (**a, b**) Electrophoresis chromatography showing PCR amplification level with different DNA polymerase products from templates with or without mismatch base, forward and reverse watching primer respectively. (**c**) Sanger sequencing chromatography of PCR products from **a** and **b**. (**d, e**) Bar chart illustrating sensitivity of multiple qPCR products to single-base mismatch at different position relative to 3' end, with forward and reverse watching primer respectively. (Means ± s.e.m, n=3 independent technical replicates)

Fig. S3

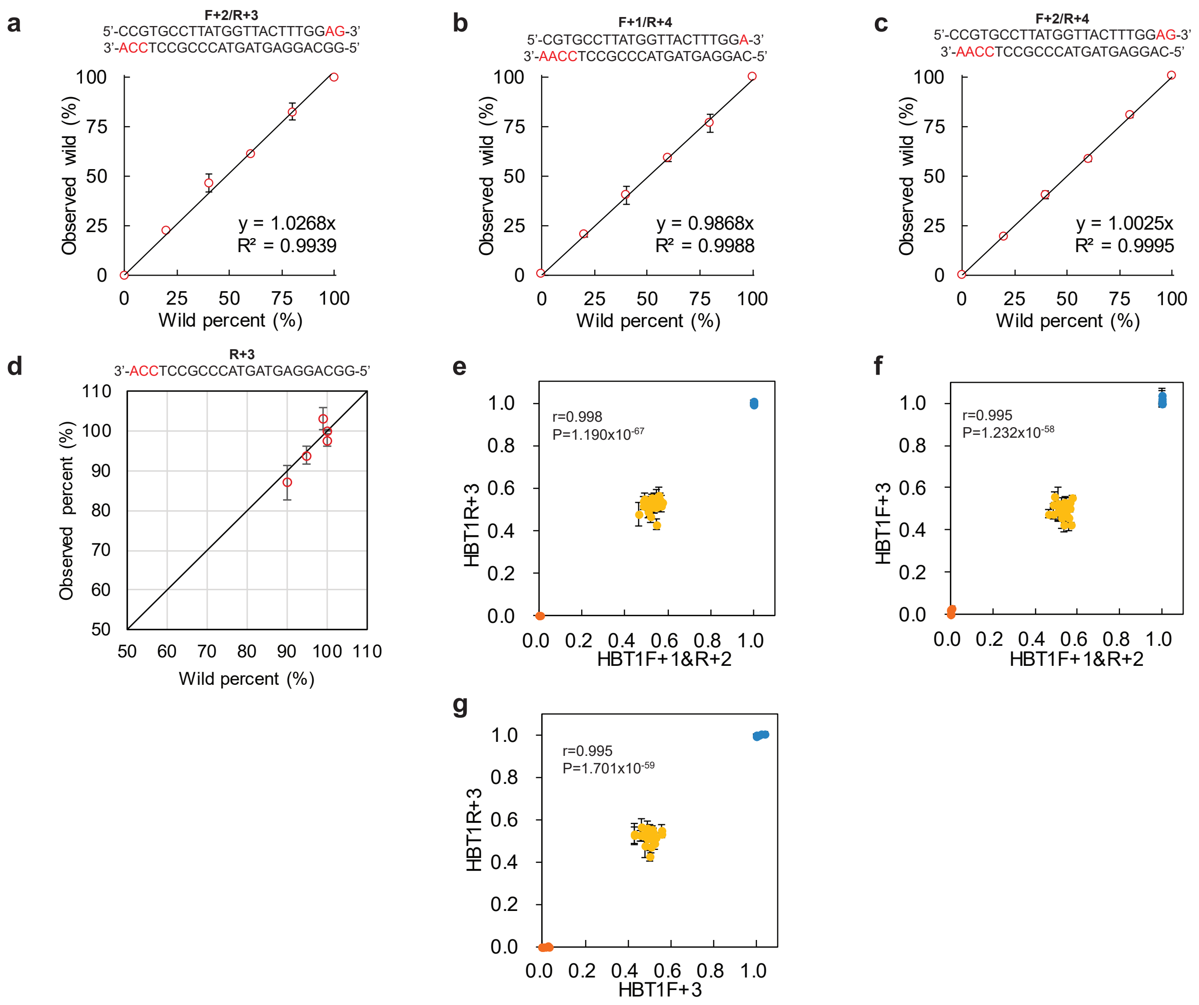

Supplementary Figure 3 Editing frequency determination and single-cell colony genotyping with indel mimic constructs. (**a-c**) Twenty-six plasmids simulating indels at HOXB13 gene target 1 were mixed with wild type HOXB13 plasmid at given ratios and evaluated by getPCR method using forward and reverse watching primer in combination. (**d**) Plasmid indel mimics at *HOXB13* gene target 1with wild percent of 90%, 95%, 99% and 99.9% were subjected to getPCR evaluation. Watching primer is shown on the top of panel. (Means ± s.e.m, n=3 independent technical replicates) (**f-g**) Genotyping of mimic single-cell clones by combination of two differently designed getPCR watching primers. Referring to Figure 2a for information of mimic indels.

Fig. S4

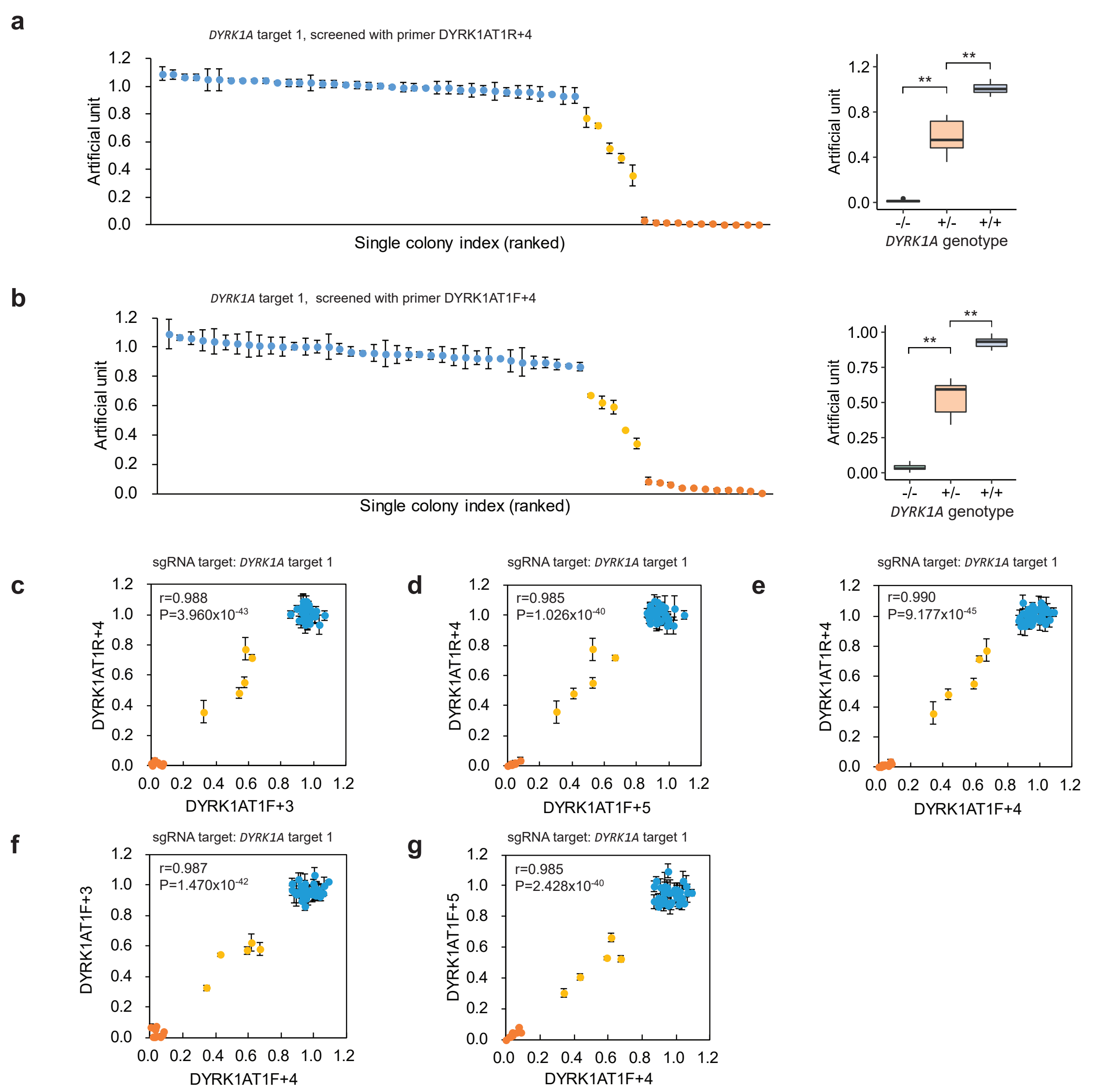

h

HOXB13 target 2

```
5’..GCTCCCGTGCCTTATGGTTACTT---TGGAGGCGGGTACTACTCCTGCCGAG..3’ wild
5’..GCTCCCGTGCCTTATGGTTACTTA--TGGAGGCGGGTACTACTCCTGCCGAG..3’
5’..GCTCCCGTGCCTTATGGTTACTTT--TGGAGGCGGGTACTACTCCTGCCGAG..3’
5’..GCTCCCGTGCCTTATGGTTACTTG--TGGAGGCGGGTACTACTCCTGCCGAG..3’
5’..GCTCCCGTGCCTTATGGTTACTTCT-TGGAGGCGGGTACTACTCCTGCCGAG..3’
5’..GCTCCCGTGCCTTATGGTTACTTGG-TGGAGGCGGGTACTACTCCTGCCGAG..3’
5’..GCTCCCGTGCCTTATGGTTACTTTT-TGGAGGCGGGTACTACTCCTGCCGAG..3’
5’..GCTCCCGTGCCTTATGGTTACTTCTTTGGAGGCGGGTACTACTCCTGCCGAG..3’
5’..GCTCCCGTGCCTTATGGTTACT----TGGAGGCGGGTACTACTCCTGCCGAG..3’
5’..GCTCCCGTGCCTTATGGTTACT-----GGAGGCGGGTACTACTCCTGCCGAG..3’
5’..GCTCCCGTGCCTTATGGTTAC------GGAGGCGGGTACTACTCCTGCCGAG..3’
5’..GCTCCCGTGCCTTATGGTTAC--CATA--AGGCGGGTACTACTCCTGCCGAG..3’
5’..GCTCCCGTGCCTTATGGT-AC---------GGCGGGTACTACTCCTGCCGAG..3’
5’..GCTCCCGTGCCTTATGG------------AGGCGGGTACTACTCCTGCCGAG..3’
5’..GCTCCCGTG-------------------GAGGCGGGTACTACTCCTGCCGAG..3’
5’..--------------------------TGGAGGCGGGTACTACTCCTGCCGAG..3’
5’..GCTCCCGTGCCTTA------------TGGAGGCGGGTACTACTCCTGCCGAG..3’
5’..GCTCCCGTGCCTTATGGT---------GGAGGCGGGTACTACTCCTGCCGAG..3’
5’..GCTCCCGTGCCTTATGGTTAC----------GCGGGTACTACTCCTGCCGAG..3’
5’..GCTCCCGTGCCTTATGGTTACTT--------GCGGGTACTACTCCTGCCGAG..3’
5’..GCTCCCGTGCCTTATGGTTAC------------GGGTACTACTCCTGCCGAG..3’
5’..GCTCCCGTGCCTTATGG---C--------A--C-GGTACTACTCCTGCCGAG..3’
5’..GCTCCCGTGCCTTATGGT------------------TACTACTCCTGCCGAG..3’
5’..GCTCCCGTGCCTTATGGTTACTT----G---------ACTACTCCTGCCGAG..3’
5’..GCTCCCGTGCCTTATGGTTACTT---------------CTACTCCTGCCGAG..3’
5’..GCTCCCGTGCCTTATGGT---------------------TACTCCTGCCGAG..3’
5’..GCTCCCGTGCCTTATGGTTACTT-----------------ACTCCTGCCGAG..3’
5’..GCTCCCGTGCCTTATGGTTA------------------------CTGCCGAG..3’
5’..GCTCCCGTGCCTTATGGTTACTT-------------------------CGAG..3’
5’..GCTCCCGTGCCTTATGGTT---------------------------------..3’
5’..GCTCCCGTGCC---------------------------------CTGCCGAG..3’
```

i

EMX1 target 5

```
5’..CATCGATGTCACCTCCAATGACTA---GGGTGGGCAACCACAAACCCACGA..3’ wild
5’..CATCGATGTCACCTCCAATGACTATA-GGGTGGGCAACCACAAACCCACGA..3’
5’..CATCGATGTCACCTCCAATGACTA----GGTGGGCAACCACAAACCCACGA..3’
5’..CATCGATGTCACCTCCAATGACTAAGTTTGTGGGCAACCACAAACCCACGA..3’
5’..CATCGATGTCACCTCCAATGAC--AT----TGGGCAACCACAAACCCACGA..3’
5’..CATCGATGTCACCTCCAAT----------GTGGGCAACCACAAACCCACGA..3’
5’..CATCGATGTCACCTCCAATG----TA-GGGTGGGCAACCACAAACCCACGA..3’
5’..CATCGATGTCACCTCCAATGA-------------CAACCACAAACCCACGA..3’
5’..CATCGATGTCACCTCCAATGA-------GGTG-------------CCACGA..3’
5’..CATCGATGTCACCTCCAA-------------------CCACAAACCCACGA..3’
5’..CATCGATGTCACCT-------------------------ACAAACCCACGA..3’
5’..CATCGATGTCACCT-----------------------CCACAAACCCACGA..3’
5’..CATCGATGTCACCT---ATCCC----------GGCAACCACAAACCCACGA..3’
5’..CATCGATGTCACC-AC---GT------GGGTGGGCAACCACAAACCCACGA..3’
5’..----------------------------------CAACCACAAACCCACGA..3’
5’..--T-GA---CA--TC----GA-T------GT-------CACAAACCCACGA..3’
5’..---------------------------------------------------..3’
```

j

DYRK1A target 1

```
5’..TCCATGCTGCTGGCCTTCAGAT-GGCTGGACAGATGCCCCATTCACA..3’ wild
5’..TCCATGCTGCTGGCCTTCAGATTGGCTGGACAGATGCCCCATTCACA..3’
5’..TCCATGCTGCTGGCCTTCAGAT---------AGATGCCCCATTCACA..3’
5’..TCCATGCTGCTGGCCTTCA---------GACAGATGCCCCATTCACA..3’
5’..TCCATGCTGCTGGCCTTCA-ACATGCT-GACAGATGCCCCATTCACA..3’
5’..TCCATGCTGCTGGCCTTCA----GGCTGGACAGATGCCCCATTCACA..3’
5’..TCCATGCTGCTGGCCTT-------------CAGATGCCCCATTCACA..3’
5’..TCCATGCTGCTGGC------------TGGACAGATGCCCCATTCACA..3’
5’..TCCATGCTGCT---------AT-GGCTGGACAGATGCCCCATTCACA..3’
5’..TCCATGCT---------------GGCTGGACAGATGCCCCATTCACA..3’
5’..TCCATGCT----------------GCTGGACAGATGCCCCATTCACA..3’
5’..---------------------------------ATGCCCCATTCACA..3’
```

k

EMX1 target 1

```
5’..CCTATGTAGCCTCAGTCTTCCC-ATCAGGCTCTCAGCTCAGCCTGAGT..3’ wild
5’..CCTATGTAGCCTCAGTCTTCCCCATCAGGCTCTCAGCTCAGCCTGAGT..3’
5’..CCTATGTAGCCTCAG--------ATCAGGCTCTCAGCTCAGCCTGAGT..3’
5’..CCTATGTAGC-TGA--------------GCTCTCAGCTCAGCCTGAGT..3’
5’..CCTATGTAGCCTCAGTCTTCCC--T----CTCTCAGCTCAGCCTGAGT..3’
5’..CCTATGTAGCCTCAGTCTTCCC--------TCTCAGCTCAGCCTGAGT..3’
5’..CCTATGTAGCCTCAGTCTTC------------TCAGCTCAGCCTGAGT..3’
5’..CCTATGTAGCCTCAGTCT--------------TCAGCTCAGCCTGAGT..3’
5’..CCTATGTAGC---------------------------TCAGCCTGAGT..3’
5’..CCTATGTAGCCTCA--------------------------GCCTGAGT..3’
5’..CCTATGTAGCCTCAG--------------------------CCTGAGT..3’
5’..CCTATGTAGCCTCAGTCTTCCC---------------------TGAGT..3’
5’..CCTATGTAGCCTCAGTCTT-----------------------------..3’
5’..------------------------------------------------..3’
```

Supplementary Figure 4 Single-cell colony genotyping for indels induced by gRNAs targeting *HOXB13*, *DYRK1A* and *EMX1* genes. (**a, b**) Genotyping of single-cell clones coming from edited 293T cells targeting *DYRK1A* gene through getPCR method with two differently designed watching primer respectively. Box plots show quartiles with a band at median, whiskers indicating 1.5 IQR, and outliers shown separately. (**c-g**) Scatterplots showing the correlation and combination effect of two differently designed watching primers in genotyping. (**h-k**) Illustration of indels discovered in single-cell clone genotyping by Sanger sequencing, for gRNA *HOXB13* target 2, *EMX1* target 5, *DYRK1A* target1 and *EMX1* target1 respectively. (Means ± s.e.m, n=3 independent technical replicates, *P < 0.05, **P < 0.01, ***P < 0.001)

Fig. S5

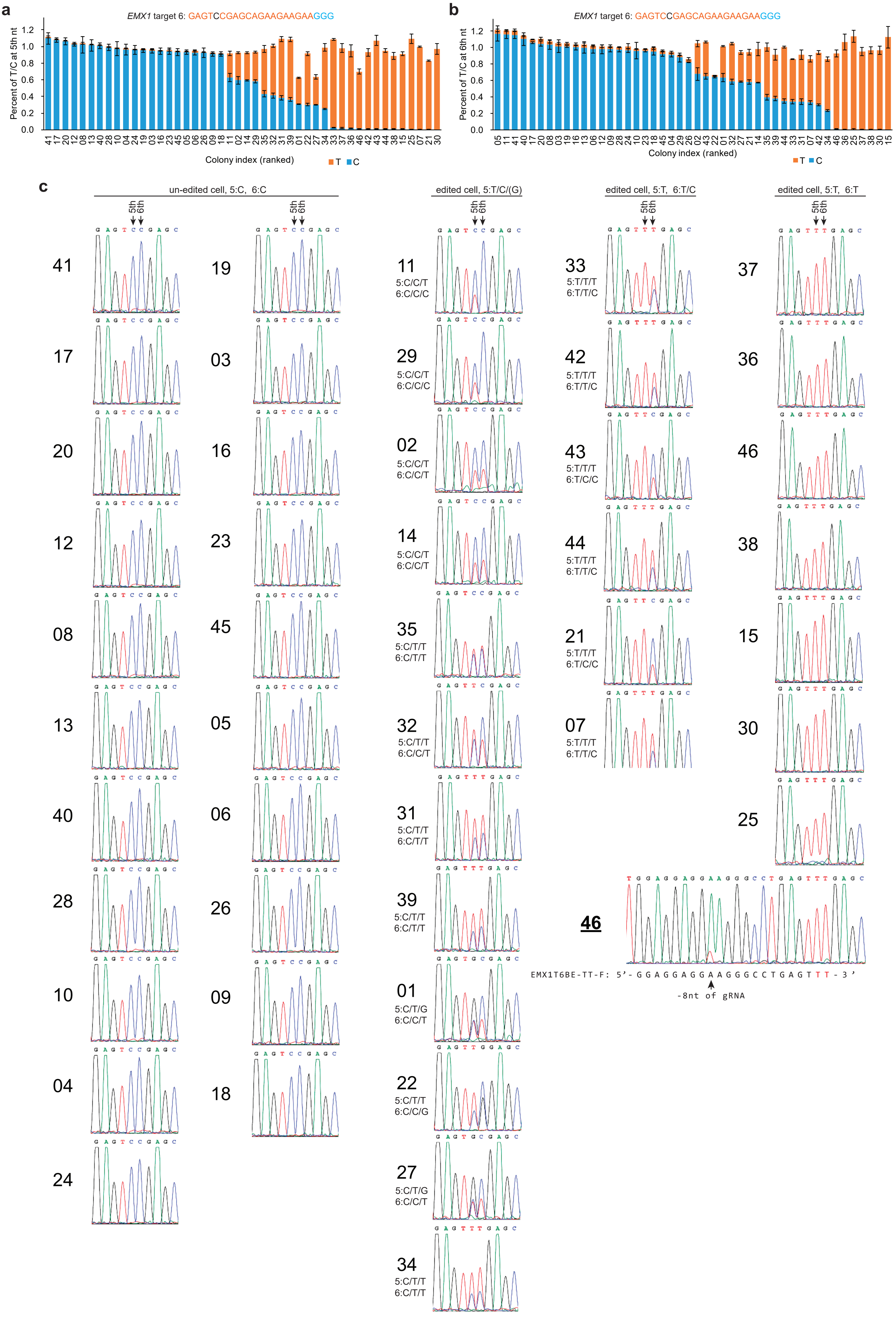
a
EMX1 target 6: GAGTCCGAGCAGAAGAAGAAGGG
Percent of T/C at 5th nt
Colony index (ranked)
T C
b
EMX1 target 6: GAGTCCGAGCAGAAGAAGAAGGG
Percent of T/C at 6th nt
Colony index (ranked)
T C
c
un-edited cell, 5:C, 6:C
edited cell, 5:T/C/(G)
edited cell, 5:T, 6:T/C
edited cell, 5:T, 6:T
5th 6th
41
17
20
12
08
13
40
28
10
04
24
19
03
16
23
45
05
06
26
09
18
11 5:C/C/T 6:C/C/C
29 5:C/C/T 6:C/C/C
02 5:C/C/T 6:C/C/T
14 5:C/C/T 6:C/C/T
35 5:C/T/T 6:C/T/T
32 5:C/T/T 6:C/C/T
31 5:C/T/T 6:C/T/T
39 5:C/T/T 6:C/T/T
01 5:C/T/G 6:C/C/T
22 5:C/T/T 6:C/C/G
27 5:C/T/G 6:C/C/T
34 5:C/T/T 6:C/T/T
33 5:T/T/T 6:T/T/C
42 5:T/T/T 6:T/T/C
43 5:T/T/T 6:T/C/C
44 5:T/T/T 6:T/T/C
21 5:T/T/T 6:T/C/C
07 5:T/T/T 6:T/T/C
37
36
46
38
15
30
25
46
EMX1T6BE-TT-F: 5'-GGAGGAGGAAGGGCCTGAGTTT-3'
-8nt of gRNA

Supplementary Figure 5 Single-cell colony genotyping in base editing by gRNAs targeting *EMX1* gene. (**a**) Bar chart showing single-cell clone genotyping at 5th nucleotide by getPCR in *EMX1* gene base editing experiment, i.e., **figure 6j** annotated with detailed clone number. (**b**) Bar chart showing single-cell clone genotyping at 6th nucleotide by getPCR in *EMX1* gene base editing experiment, i.e., **figure 6l** annotated with detailed clone number. (**c**) Sanger sequencing chromatography in genotyping of single-cell clone. (Means ± s.e.m, n=3 independent technical replicates)

Fig. S6

a

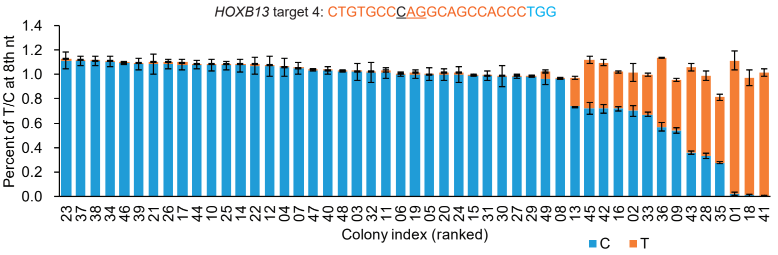
HOXB13 target 4: CTGTGCCCAGGCAGCCACCCTGG
Percent of T/C at 8th nt
1.4
1.2
1.0
0.8
0.6
0.4
0.2
0.0
23 37 38 34 46 39 21 26 17 44 10 25 14 22 12 04 07 47 40 48 03 32 11 06 19 05 20 24 15 31 30 27 29 49 08 13 45 42 16 02 33 36 09 43 28 35 01 18 41
Colony index (ranked)
C
T

b

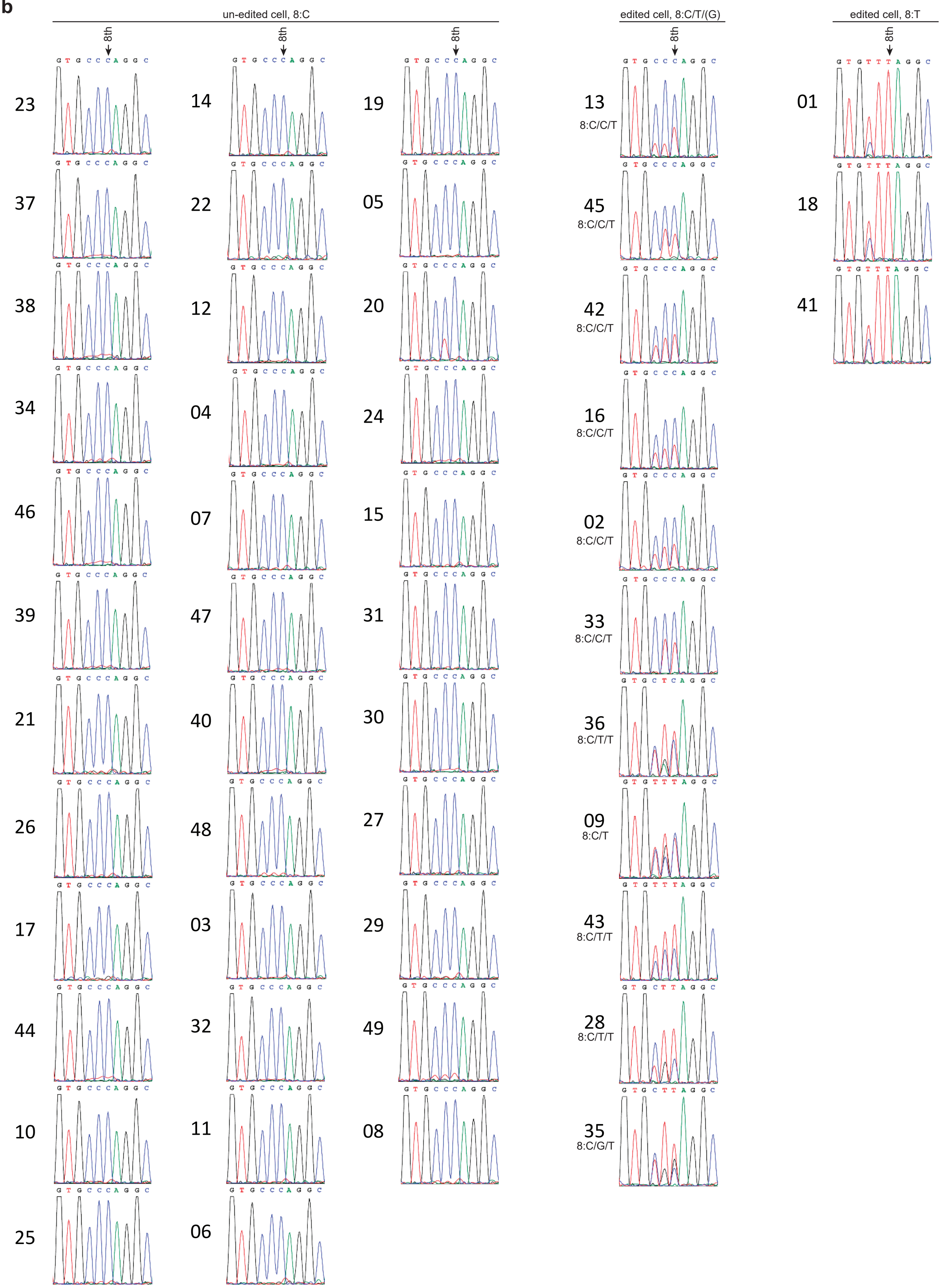
un-edited cell, 8:C
edited cell, 8:C/T/(G)
edited cell, 8:T
8th
23
37
38
34
46
39
21
26
17
44
10
25
14
22
12
04
07
47
40
48
03
32
11
06
19
05
20
24
15
31
30
27
29
49
08
13
8:C/C/T
45
8:C/C/T
42
8:C/C/T
16
8:C/C/T
02
8:C/C/T
33
8:C/C/T
36
8:C/T/T
09
8:C/T
43
8:C/T/T
28
8:C/T/T
35
8:C/G/T
01
18
41

Supplementary Figure 6 Single-cell colony genotyping for base-editing introduced stop codon on *HOXB13* gene. (**a**) Bar chart showing single-cell clone genotyping at 8th nucleotide by getPCR in *HOXB13* gene base editing experiment, i.e., **figure 6n** annotated with detailed clone number. (**b**) Sanger sequencing chromatography in genotyping of single-cell clone. (Means ± s.e.m, n=3 independent technical replicates)

Fig. S7

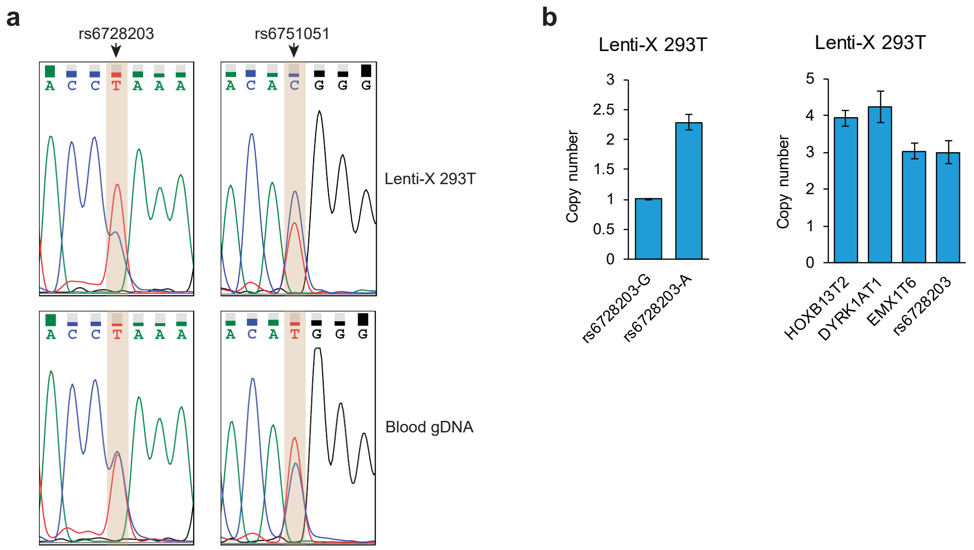

Supplementary Figure 7. Triploid character of Lenti-X 293T at given region. (**a**) Sanger sequencing chromatography of rs6728203 and rs6751051 region ('-'strand) in Lenti-X 293T and blood genomic DNA of one author. (**b**) Gene copy number evaluation of *EMX1*, *HOXB13* and *DYRK1A* by real time PCR using primers targeting the given gRNA region. The G allele of rs6728203 was used for calibration referernce of Lenti-X 293T to blood genomic DNA.

Table S1. oligos used in plasmid construction and transfection

a. Primers for construction of indel-mimic HOXB13 variant plasmid through site-directed mutagenesis

Pladmid	Primer 1 (5'→3')	Primer 2 (5'→3')	Template	
HB251d1L	aggcgggtactactcctgc	caaagtaaccataaggcacgg	OE1	
HB251d2L	aggcgggtactactcctgc	aaagtaaccataaggcacggg	OE1	
HB251d3L	aggcgggtactactcctgc	aagtaaccataaggcacggga	OE1	
HB251d5L	aggcgggtactactcctgc	gtaaccataaggcacgggagc	OE1	
HB251d10L	aggcgggtactactcctgc	cataaggcacgggagctgg	OE1	
HB251d15L	aggcgggtactactcctgc	ggcacgggagctggggacg	OE1	
HB251d1R	ggcgggtactactcctgcc	ccaaagtaaccataaggcacg	OE1	
HB251d2R	gcgggtactactcctgccg	ccaaagtaaccataaggcacg	OE1	
HB251d3R	cgggtactactcctgccga	ccaaagtaaccataaggcacg	OE1	got HB251d8R unexpectedly
HB251d5R	ggtactactcctgccgagtgt	ccaaagtaaccataaggcacg	OE1	
HB251d10R	tactcctgccgagtgtccc	ccaaagtaaccataaggcacg	OE1	
HB251d15R	ctgccgagtgtcccggagc	ccaaagtaaccataaggcacg	OE1	
HB251d1L1R	ggcgggtactactcctgcc	caaagtaaccataaggcacgg	OE1	
HB251d2L2R	gcgggtactactcctgccg	aaagtaaccataaggcacggg	OE1	
HB251d2L3R	cgggtactactcctgccga	aaagtaaccataaggcacggg	OE1	
HB251d2L5R	ggtactactcctgccgagtgt	aaagtaaccataaggcacggg	OE1	
HB251d2L10R	tactcctgccgagtgtccc	aaagtaaccataaggcacggg	OE1	
HB251iA	aaggcgggtactactcctgc	ccaaagtaaccataaggcacg	OE1	
HB251iT	taggcgggtactactcctgc	ccaaagtaaccataaggcacg	OE1	
HB251iG	gaggcgggtactactcctgc	ccaaagtaaccataaggcacg	OE1	
HB251iC	caggcgggtactactcctgc	ccaaagtaaccataaggcacg	OE1	
HB251iAA	aaaggcgggtactactcctgc	ccaaagtaaccataaggcacg	OE1	
HB251iAT	ataggcgggtactactcctgc	ccaaagtaaccataaggcacg	OE1	
HB251iAG	agaggcgggtactactcctgc	ccaaagtaaccataaggcacg	OE1	
HB251iAC	acaggcgggtactactcctgc	ccaaagtaaccataaggcacg	OE1	
HB251iAAA	aaaaggcgggtactactcctgc	ccaaagtaaccataaggcacg	OE1	
1MM-G	ggcgggtactactcctgccg	cccaaagtaaccataaggcacg	OE1	
2MM-G	ggcgggtactactcctgccg	ctcaaagtaaccataaggcacg	OE1	
3MM-G	ggcgggtactactcctgccg	cttaaagtaaccataaggcacg	OE1	
1MM-C	cgcgggtactactcctgccg	cccaaagtaaccataaggcacg	OE1	
2MM-C	cgcgggtactactcctgccg	ctcaaagtaaccataaggcacg	OE1	
3MM-C	cgcgggtactactcctgccg	cttaaagtaaccataaggcacg	OE1	
1MM-A	tgcgggtactactcctgccg	cccaaagtaaccataaggcacg	OE1	
2MM-A	tgcgggtactactcctgccg	ctcaaagtaaccataaggcacg	OE1	
3MM-A	tgcgggtactactcctgccg	cttaaagtaaccataaggcacg	OE1	
1MM-T	agcgggtactactcctgccg	cccaaagtaaccataaggcacg	OE1	
2MM-T	agcgggtactactcctgccg	ctcaaagtaaccataaggcacg	OE1	
3MM-T	agcgggtactactcctgccg	cttaaagtaaccataaggcacg	OE1	
A252T	cttatggttactttggTggcgggtactactcc	ggagtagtacccgccaccaaagtaaccataag	OE1	
G253C	cttatggttactttggAcgcgggtactactcc	ggagtagtacccgcgtccaaagtaaccataag	1MM-C	
G253A	cttatggttactttggAagcgggtactactcc	ggagtagtacccgcttccaaagtaaccataag	1MM-A	

b. Primers for construction of blank sgRNA expression plasmid

Name	Primer 1 (5'→3')	Primer 2 (5'→3')	
sgRNA expression plasmid	tccgggagctgcatgtgtca	gggtacctctagagccatttg	addgene#42230

c. Primers for construction of HF-Cas9(R661A, Q695A, Q926A) through site-directed mutagenesis

Cas9 variation	Primer 1 (5'→3')	Primer 2 (5'→3')
Cas9R661A	accggctggggcgcgctgagccggaag	cttccggctcagcgcgccccagccggt
Cas9Q695A	[illegible]aacagaaacttcatggcgctgatccacgacga[illegible]	gtcgtcgtggatcagcgccatgaagtttctgttgg
Cas9Q926A	[illegible]tggtggaaacccgggcgatcacaaagcacgt[illegible]	cacgtgctttgtgatcgcccgggtttccaccag

d. Primers for construction of sgRNA expression plasmids of given targets

Target	Primer 1 (5'→3')	Primer 2 (5'→3')
HOXB13 target 1	caccgccttatggttactttggagg	aaaccctccaaagtaaccataaggc
HOXB13 target 2	caccgtgccttatggttactttgg	aaacccaaagtaaccataaggcac
HOXB13 target 3	caccgccataggctggtaggttcc	aaacggaacctaccagcctatggc
HOXB13 target 4	caccgctgtgcccaggcagccaccc	aaacgggtggctgcctgggcacagc
DYRK1A target 1	caccggctgctggccttcagatggc	aaacgccatctgaaggccagcagcc
EMX1 target 1	caccggtagcctcagtcttcccatc	aaacgatgggaagactgaggctacc
EMX1 target 2	caccggaggccccagtggctgctct	aaacagagcagccactggggcctcc
EMX1 target 3	caccggggcaaccacaaacccacga	aaactcgtgggtttgtggttgcccc
EMX1 target 4	caccgggcagagtgctgcttgctgc	aaacgcagcaagcagcactctgccc
EMX1 target 5	caccggtcacctccaatgactaggg	aaaccccctagtcattggaggtgacc
EMX1 target 6	caccggagtccgagcagaagaagaa	aaacttcttcttctgctcggactcc

e. HDR template sequence (5'→3')

EMX1-HindIII	cacgaagcaggccaatggggaggacatcgatgtcacctccaatgactAAGCTTgggcaacca caaacccacgagggcagagtgctgcttgctgctggcc

Table S2. Oligos for genome editing efficiency determination on indel-mimic plasmids using getPCR and Surveyor

a. Primers for Surveyor DNA amplification and sanger sequencing

Gene	Primer 1 (5'→3')	Primer 2 (5'→3')
HOXB13	ccggcaattatgccaccttg	ggtgggttctgttctccctg
DYRK1A	ggagctggtctgttggagaa	tcccaatccataatcccacgtt
EMX1	ccatccccttctgtgaatgt	ggagattggagacacggaga

b. Primers for getPCR in detecting indels at HOXB13 target 1 site

Watching Primer ID	Watching primer (5'→3')	paired primer (5'→3')
R+1	gcaggagtagtacccgcctc	cctggggtgccccaggggac
R+2	caggagtagtacccgcctcc	cctggggtgccccaggggac
R+3	caggagtagtacccgcctcca	cctggggtgccccaggggac
R+4	caggagtagtacccgcctccaa	cctggggtgccccaggggac
R+5	aggagtagtacccgcctccaaa	cctggggtgccccaggggac
R+6	ggagtagtacccgcctccaaag	cctggggtgccccaggggac
R+7	ggagtagtacccgcctccaaagt	cctggggtgccccaggggac
R+8	ggagtagtacccgcctccaaagta	cctggggtgccccaggggac
F+1	cccgtgccttatggttactttgga	ggggcggctggggtactcttc
F+2	ccgtgccttatggttactttggag	ggggcggctggggtactcttc
F+3	cgtgccttatggttactttggagg	ggggcggctggggtactcttc
F+4	gtgccttatggttactttggaggc	ggggcggctggggtactcttc
F+5	gccttatggttactttggaggcg	ggggcggctggggtactcttc
F+6	gccttatggttactttggaggcgg	ggggcggctggggtactcttc
F+7	cttatggttactttggaggcggg	ggggcggctggggtactcttc
F+8	cttatggttactttggaggcgggt	ggggcggctggggtactcttc
F+3-1	tgccttatggttactttggagg	ggggcggctggggtactcttc
F+3-2	gtgccttatggttactttggagg	ggggcggctggggtactcttc
F+3-3	cgtgccttatggttactttggagg	ggggcggctggggtactcttc
F+3-4	ccgtgccttatggttactttggagg	ggggcggctggggtactcttc
F+3-5	cccgtgccttatggttactttggagg	ggggcggctggggtactcttc
F+4-1	gccttatggttactttggaggc	ggggcggctggggtactcttc
F+4-2	tgccttatggttactttggaggc	ggggcggctggggtactcttc
F+4-3	gtgccttatggttactttggaggc	ggggcggctggggtactcttc
F+4-4	cgtgccttatggttactttggaggc	ggggcggctggggtactcttc
F+4-5	ccgtgccttatggttactttggaggc	ggggcggctggggtactcttc
R+4-1	ggagtagtacccgcctccaa	cctggggtgccccaggggac
R+4-2	aggagtagtacccgcctccaa	cctggggtgccccaggggac
R+4-3	caggagtagtacccgcctccaa	cctggggtgccccaggggac
R+4-4	gcaggagtagtacccgcctccaa	cctggggtgccccaggggac
R+4-5	ggcaggagtagtacccgcctccaa	cctggggtgccccaggggac
R+3-1	ggagtagtacccgcctcca	cctggggtgccccaggggac
R+3-2	aggagtagtacccgcctcca	cctggggtgccccaggggac
R+3-3	caggagtagtacccgcctcca	cctggggtgccccaggggac
R+3-4	gcaggagtagtacccgcctcca	cctggggtgccccaggggac
R+3-5	ggcaggagtagtacccgcctcca	cctggggtgccccaggggac
253-G	cccgtgccttatggttactttggag	cagtggggcggctggggta
253-C	cccgtgccttatggttactttggac	cagtggggcggctggggta
253-A	cccgtgccttatggttactttggaa	cagtggggcggctggggta
253-T	cccgtgccttatggttactttggat	cagtggggcggctggggta

Table S3. Oligos for in-cell genome editing efficiency quantification by getPCR

a. getPCR primers for indel efficiency quantification

Target site	Watching primer	Watching primer (5'→3')	paired primer (5'→3')
HOXB13 target 2	HOXB13T2F+3	tcccgtgccttatggttactttgg	cagtggggcggctggggta
HOXB13 target 2	HOXB13T2F+4	tcccgtgccttatggttactttgga	cagtggggcggctggggta
HOXB13 target 2	HOXB13T2R+3	ggagtagtacccgcctccaaag	tggggtgccccaggggac
HOXB13 target 2	HOXB13T2R+4	ggagtagtacccgcctccaaagt	tggggtgccccaggggac
HOXB13 target 3	HOXB13T3F+3	ctatccgggatatccgggaacc	gagtctgcaccacagacacgtcc
HOXB13 target 3	HOXB13T3F+4	ctatccgggatatccgggaacct	gagtctgcaccacagacacgtcc
HOXB13 target 3	HOXB13T3R+3	tggccataggctggtaggttcc	accccgcggagactcccacg
HOXB13 target 3	HOXB13T3R+5	ccataggctggtaggttcccg	accccgcggagactcccacg
	**Control Primers**	**Primer 1 (5'→3')**	**Primer 2 (5'→3')**
	HOXB13-104ctrl	gcgacatgactccctgttgcctgtg	gacctggtgggttctgttctccctg

Target site	Watching primer	Watching primer (5'→3')	paired primer (5'→3')
DYRK1A target 1	DYRK1AT1R+4	tggggcatctgtccagccatct	ttgtaggaggagagacttcagcatgc
DYRK1A target 1	DYRK1AT1F+3	tgctgctggccttcagatggc	tatgataaggcagaaacctgttggtcac
DYRK1A target 1	DYRK1AT1F+4	tgctgctggccttcagatggct	tatgataaggcagaaacctgttggtcac
DYRK1A target 1	DYRK1AT1F+5	ctgctggccttcagatggctg	tatgataaggcagaaacctgttggtcac
	**Control Primers**	**Primer 1 (5'→3')**	**Primer 2 (5'→3')**
	DYRK1A-99ctrl	gcgatgttgtttgccgtaaacctggc	gacttctccaacagaccagctcctc

Target site	Watching primer	Watching primer (5'→3')	paired primer (5'→3')
EMX1 target 1	EMX1T1R+4	ccatccccttctgtgaatgttagaccc	ctgagctgagagcctgatggga
EMX1 target 2	EMX1T2F+3	gaggccccagtggctgctct	ttgatgtgatgggagcccttcttct
	**Control Primers**	**Primer 1 (5'→3')**	**Primer 2 (5'→3')**
	EMX1-113ctrl	cgatgtcacctccaatgactagggtg	cagggagtggccagagtccagct

Target site	Watching primer	Watching primer (5'→3')	paired primer (5'→3')
EMX1 target 3	EMX1T3F+3	gtgggcaaccacaaacccacga	aggggcctggccagcagca
EMX1 target 3	EMX1T3R+5	agcactctgccctcgtgggt	gatgtcacctccaatgactagggt
EMX1 target 4	EMX1T4R+3	aggggcctggccagcagca	gtgggcaaccacaaacccacga
EMX1 target 4	EMX1T4F+4	ggcagagtgctgcttgctgct	tccccaaagcctggccagggagt
EMX1 target 5	EMX1T5F+4	gatgtcacctccaatgactagggt	agcactctgccctcgtgggt
EMX1 target 5	EMX1T5R+4	ggtttgtggttgcccaccctagt	gcctgagtccgagcagaagaagaa
EMX1 target 6	EMX1T6F+3	gcctgagtccgagcagaagaagaa	ggtttgtggttgcccaccctagt
EMX1 target 6	EMX1T6R+4	ttgatgtgatgggagcccttcttct	gaggccccagtggctgctct
	**Control Primers**	**Primer 1 (5'→3')**	**Primer 2 (5'→3')**
	EMX1-111ctrl	ccatccccttctgtgaatgttagaccc	tgagctgagagcctgatgggaagac

b. getPCR primers for base editing efficiency quantification

Target site	Watching primer	Watching primer (5'→3')	paired primer (5'→3')
HOXB13 target 4	C8	cggccagggtggctgcctG	ccgtgccttatggttactttggagg
HOXB13 target 4	T8	cggccagggtggctgcctA	ccgtgccttatggttactttggagg
	**Control Primers**	**Primer 1 (5'→3')**	**Primer 2 (5'→3')**
	HOXB13-104ctrl	gcgacatgactccctgttgcctgtg	gacctggtgggttctgttctccctg

Target site	Watching primer	Watching primer (5'→3')	paired primer (5'→3')
EMX1 target 6	5C	ggaggaggaagggcctgagtC	ggtttgtggttgcccaccctagt
EMX1 target 6	5T	tggaggaggaagggcctgagtT	ggtttgtggttgcccaccctagt
EMX1 target 6	6C	ggagcccttcttcttctgctcG	gaggccccagtggctgctct
EMX1 target 6	6T	gggagcccttcttcttctgctcA	gaggccccagtggctgctct
EMX1 target 6	CC	gaggaggaagggcctgagtCC	ggtttgtggttgcccaccctagt
EMX1 target 6	TT	ggaggaggaagggcctgagtTT	ggtttgtggttgcccaccctagt
EMX1 target 6	CT	gaggaggaagggcctgagtCT	ggtttgtggttgcccaccctagt
EMX1 target 6	TC	gaggaggaagggcctgagtTC	ggtttgtggttgcccaccctagt
	**Control Primers**	**Primer 1 (5'→3')**	**Primer 2 (5'→3')**
	EMX1-111ctrl	ccatccccttctgtgaatgttagaccc	tgagctgagagcctgatgggaagac

c. getPCR primers for HDR repairing efficiency quantification

Target site	Watching primer	Watching primer (5'→3')	paired primer (5'→3')
EMX1 target 5	EMX1T5HindIII-F	tcgatgtcacctccaatgactAAGCTT	agcactctgccctcgtgggt
EMX1 target 5	EMX1T5HindIII-R	gggtttgtggttgcccaAGCTTag	gcctgagtccgagcagaagaagaa
	**Control Primers**	**Primer 1 (5'→3')**	**Primer 2 (5'→3')**
	EMX1-111ctrl	ccatccccttctgtgaatgttagaccc	tgagctgagagcctgatgggaagac

Table S4.Oligos and program for genome editing efficiency quantification by NGS

a. Primers for library preparation

First Round

target site	Primer ID	First Round Primer 1 sequence (5’→3’)	First Round Primer 2 sequence (5’→3’)
HOXB13-T2	HB13-251lib	ACTCTTTCCCTACACGACGCTCTTCCGATCTctgtcaactatgccccttggatctg	GTGACTGGAGTTCAGACGTGTGCTCTTCCGATCTggcaaactcagtggggcggctgg
HOXB13-T3	HB13-404lib	ACTCTTTCCCTACACGACGCTCTTCCGATCTcccggagctcgctgaaaccctgtg	GTGACTGGAGTTCAGACGTGTGCTCTTCCGATCTgttccagccaccagcgagagccca
HOXB13-T4 (BE4)	HB13-251lib	ACTCTTTCCCTACACGACGCTCTTCCGATCTctgtcaactatgccccttggatctg	GTGACTGGAGTTCAGACGTGTGCTCTTCCGATCTggcaaactcagtggggcggctgg
DYRK1A-T1	DYRK1Alib	ACTCTTTCCCTACACGACGCTCTTCCGATCTggatatgaatatttcctttaaacctcac	GTGACTGGAGTTCAGACGTGTGCTCTTCCGATCTtccatgaacttacctggttagttag
EMX1-T1	EMX1-1lib	ACTCTTTCCCTACACGACGCTCTTCCGATCTccatccccttctgtgaatgttagacc	GTGACTGGAGTTCAGACGTGTGCTCTTCCGATCTccttcctcctccagcttctgccgt
EMX1-T2	EMX1-1lib	ACTCTTTCCCTACACGACGCTCTTCCGATCTccatccccttctgtgaatgttagacc	GTGACTGGAGTTCAGACGTGTGCTCTTCCGATCTccttcctcctccagcttctgccgt
EMX1-T3	EMX1-2lib	ACTCTTTCCCTACACGACGCTCTTCCGATCTggttccagaaccggaggacaaagtac	GTGACTGGAGTTCAGACGTGTGCTCTTCCGATCTgactccaggcctccccaaagcctg
EMX1-T4	EMX1-2lib	ACTCTTTCCCTACACGACGCTCTTCCGATCTggttccagaaccggaggacaaagtac	GTGACTGGAGTTCAGACGTGTGCTCTTCCGATCTgactccaggcctccccaaagcctg
EMX1-T5	EMX1-2lib	ACTCTTTCCCTACACGACGCTCTTCCGATCTggttccagaaccggaggacaaagtac	GTGACTGGAGTTCAGACGTGTGCTCTTCCGATCTgactccaggcctccccaaagcctg
EMX1-T6	EMX1-2lib	ACTCTTTCCCTACACGACGCTCTTCCGATCTggttccagaaccggaggacaaagtac	GTGACTGGAGTTCAGACGTGTGCTCTTCCGATCTgactccaggcctccccaaagcctg
EMX1-T6 (BE4)	EMX1-2lib	ACTCTTTCCCTACACGACGCTCTTCCGATCTggttccagaaccggaggacaaagtac	GTGACTGGAGTTCAGACGTGTGCTCTTCCGATCTgactccaggcctccccaaagcctg
EMX1-T5 (HDR with HindIII)	EMX1-3lib	ACTCTTTCCCTACACGACGCTCTTCCGATCTgtggttccagaaccggaggacaaagta	GTGACTGGAGTTCAGACGTGTGCTCTTCCGATCTctccaggcctccccaaagcctggc

1st round PCR: 50ng gDNA as template, 28 cycles, 15μl system, anealed @60°C, using PrimeSTAR® Max DNA Polymerase (TaKaRa

Second Round

Primer ID	Primer sequence (5’→3’)
libUni-F-universa	AATGATACGGCGACCACCGAGATCTACACACACTCTTTCCCTACACGACGCTCTTCCGA
libUni-D701-R	CAAGCAGAAGACGGCATACGAGAT**CGAGTAAT**GTGACTGGAGTTCAGACGTGTGCTCTT
libUni-D702-R	CAAGCAGAAGACGGCATACGAGAT**TCTCCGGA**GTGACTGGAGTTCAGACGTGTGCTCTT
libUni-D703-R	CAAGCAGAAGACGGCATACGAGAT**AATGAGCG**GTGACTGGAGTTCAGACGTGTGCTCTT
libUni-D704-R	CAAGCAGAAGACGGCATACGAGAT**GGAATCTC**GTGACTGGAGTTCAGACGTGTGCTCTT
libUni-D705-R	CAAGCAGAAGACGGCATACGAGAT**TTCTGAAT**GTGACTGGAGTTCAGACGTGTGCTCTT
libUni-D706-R	CAAGCAGAAGACGGCATACGAGAT**ACGAATTC**GTGACTGGAGTTCAGACGTGTGCTCTT
libUni-D707-R	CAAGCAGAAGACGGCATACGAGAT**AGCTTCAG**GTGACTGGAGTTCAGACGTGTGCTCTT
libUni-D708-R	CAAGCAGAAGACGGCATACGAGAT**GCGCATTA**GTGACTGGAGTTCAGACGTGTGCTCTT
libUni-D709-R	CAAGCAGAAGACGGCATACGAGAT**CATAGCCG**GTGACTGGAGTTCAGACGTGTGCTCTT
libUni-D710-R	CAAGCAGAAGACGGCATACGAGAT**TTCGCGGA**GTGACTGGAGTTCAGACGTGTGCTCTT
libUni-D711-R	CAAGCAGAAGACGGCATACGAGAT**GCGCGAGA**GTGACTGGAGTTCAGACGTGTGCTCTT
libUni-D712-R	CAAGCAGAAGACGGCATACGAGAT**CTATCGCT**GTGACTGGAGTTCAGACGTGTGCTCTT

2nd round PCR: 1ng of purified DNA from 1st round PCR as template, 10cycles, 15μl system,anealed @65°C, using PrimeSTAR® Max DNA Polymerase (TaKaRa

b. Characteristic sequence for read counting by R program

Target	Feature	characteristic sequence for counting
HOXB13 target 2	Wild Type	ttactttggagg
HOXB13 target 3	Wild Type	tccgggaaccta
DYRK1A target 1	Wild Type	tcagatggctgg
EMX1 target 1	Wild Type	cttcccatcagg
EMX1 target 2	Wild Type	ggctgctctggg
EMX1 target 3	Wild Type	aacccacgaggg
EMX1 target 4	Wild Type	gcttgctgctgg
EMX1 target 5	Wild Type	tgactagggtgg
EMX1 target 6	Wild Type	gaagaagaaggg
EMX1 target 5	HindIII	actAAGCTTggg
HOXB13 target 4	C8	gcggccagggtggctgcctG
HOXB13 target 4	T8	gcggccagggtggctgcctA
EMX1 target 6	T5	gaggaggaagggcctgagtT
EMX1 target 6	C5	gaggaggaagggcctgagtC
EMX1 target 6	T6	Tgagcagaagaagaagggct
EMX1 target 6	C6	Cgagcagaagaagaagggct
EMX1 target 6	C5C6	aggaggaagggcctgagtCC
EMX1 target 6	T5T6	aggaggaagggcctgagtTT
EMX1 target 6	C5T6	aggaggaagggcctgagtCT
EMX1 target 6	T5C6	aggaggaagggcctgagtTC

c. R Program for read counting

```
library(ShortRead)
reads=readFastq("libraryName")
reads
total_counts=length(reads)
total_counts
sequences=sread(reads)
dict=DNAStringSet(substr(sequences,1,150))
hits=vcountPattern("Wild Type characteristic sequence",dict,max.mismatch = 0,with.indels = FALSE
wild_type_counts=sum(hits)
wild_type_counts

library(ShortRead)
reads=readFastq("libraryName")
reads
total_counts=length(reads)
total_counts
sequences=sread(reads)
dict=DNAStringSet(substr(sequences,1,150))
hits=vcountPattern("expected_characteristic sequence",dict,max.mismatch = 0,with.indels = FALSE
expected_sequence_counts=sum(hits)
expected_sequence_counts
```

Table S5. Primers used at rs6728203 loci.

Target	Primer 1 (5'→3')	Primer 2 (5'→3')	Usage
6728203-1353bp	ATGTCCTCTGGGCAGGGCAC	ACCCTGGAGCCTGCTATGAC	Sanger sequencing
6728203-94bp	AGAAAGAAGAAAGAAACATCACCCACAC	GATCACAACCATAGAAAAACAAAATCTACAC	qPCR
6728203-A	GAAGAAAGAAACATCACCCACACTTTA	GATCACAACCATAGAAAAACAAAATCTACAC	qPCR
6728203-G	AGAAAGAAACATCACCCACACTTTG	GATCACAACCATAGAAAAACAAAATCTACAC	qPCR
